# Precision cooking for printed foods via multiwavelength lasers

**DOI:** 10.1038/s41538-021-00107-1

**Published:** 2021-09-01

**Authors:** Jonathan David Blutinger, Alissa Tsai, Erika Storvick, Gabriel Seymour, Elise Liu, Noà Samarelli, Shravan Karthik, Yorán Meijers, Hod Lipson

**Affiliations:** 1 Creative Machines Laboratory, Department of Mechanical Engineering, Columbia University in the City of New York, New York, NY USA; 2 Department of Food Technology, Wageningen University, Wageningen, Netherlands

**Keywords:** Engineering, Design, synthesis and processing

## Abstract

Additive manufacturing of food is a method of creating three-dimensional edible products layer-by-layer. While food printers have been in use since 2007, commercial cooking appliances to simultaneously cook and print food layers do not yet exist. A key challenge has been the spatially controlled delivery of cooking energy. Here, we explore precision laser cooking which offers precise temporal and spatial control over heat delivery and the ability to cook, broil, cut and otherwise transform food products via customized software-driven patterns, including through packaging. Using chicken as a model food, we combine the cooking capabilities of a blue laser (*λ* = 445 nm), a near-infrared (NIR) laser (*λ* = 980 nm), and a mid-infrared (MIR) laser (*λ* = 10.6 μm) to broil printed chicken and find that IR light browns more efficiently than blue light, NIR light can brown and cook foods through packaging, laser-cooked foods experience about 50% less cooking loss than foods broiled in an oven, and calculate the cooking resolution of a laser to be ~1 mm. Infusing software into the cooking process will enable more creative food design, allow individuals to more precisely customize their meals, disintermediate food supply chains, streamline at-home food production, and generate horizontal markets for this burgeoning industry.

## Introduction

Digitizing the cooking process using additive manufacturing (AM) techniques and software-controlled lasers enables temporal and spatial control of the arrangement of ingredients and the delivery of heat to raw food with millimeter precision^1–4^. Experimental food printing was introduced in 2007^5^, and while food printers have the ability to deposit materials to mm-scale resolution, they don’t yet have the ability to cook foods to this same degree of resolution. Integration of a multiwavelength laser cooker onboard a food printer can provide both penetrative heating and surface browning^6–10^, which would generate more creative food combinations and taste profiles.

Studies on the printing of various food products^11–19^ and the laser cooking of select foods^4,20–23^ exist, but none have investigated the use of lasers in the cooking of printed meats, tandem printing and cooking on a singular machine or organoleptic evaluation of laser-cooked meat. Current food printers do not allow us to print a layer of chicken, for example, and simultaneously reach both the required browning as well as adequate inside cooking temperatures amidst layers of printed carrot purée (Supplementary Fig. S1). Integration of a multiwavelength laser cooker onboard a food printer can skirt this obstacle by providing penetrative cooking and surface browning^3,6–10^ (Supplementary Video 1).

**Fig. 1:**
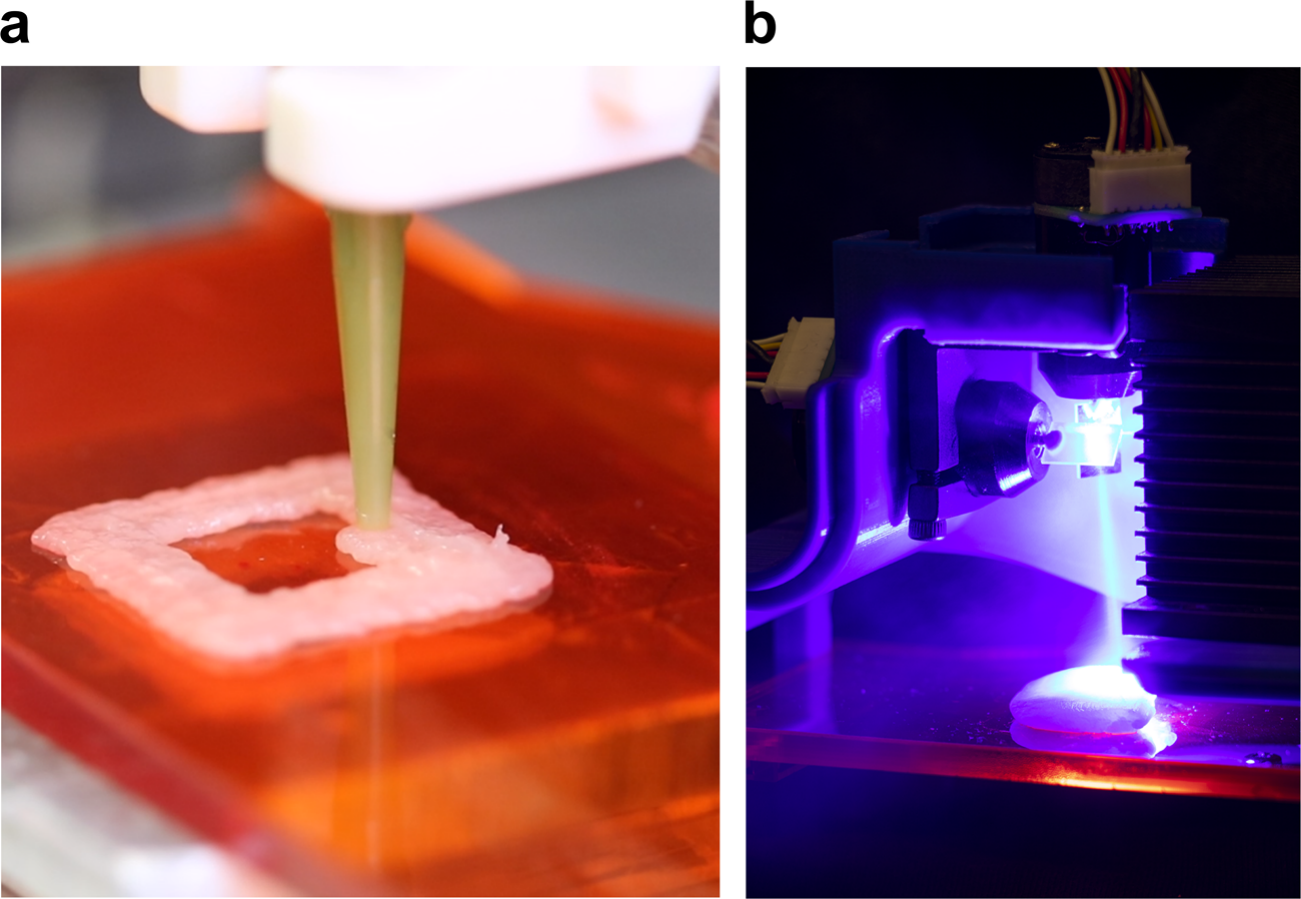
Printing and cooking apparatus. **a** Close-up of raw chicken being deposited in a square pattern from our food printer. **b** A blue laser beam being directed by a set of mirror galvanometers to a raw sample of chicken.

Here, we investigate the feasibility of printing (Fig. 1a) and cooking (Fig. 1b) food in tandem, using puréed chicken as a model food system. A 5–10 W blue diode laser (445 nm) was used as the primary heating source, but comparative tests were also done with an NIR laser (980 nm), MIR laser (10.6 μm), and a conventional toaster oven. We deduce energy required to achieve food safe temperatures in chicken; calculate cooling rates of lased chicken; investigate associated weight and volume losses with laser-broiled and oven-cooked chicken; assess color change in lased chicken; demonstrate the penetrative heating of blue and infrared light with chicken; brown chicken through plastic packaging via NIR laser (~90% transmission through plastic)^24^; and sample laser-cooked meat. Cooking trials with printed chicken samples were performed on two separate machines initially, and were then executed on the same machine. When printing and cooking were executed on the same machine, laser exposure took place after a complete layer was printed.

## Results

**Fig. 2:**
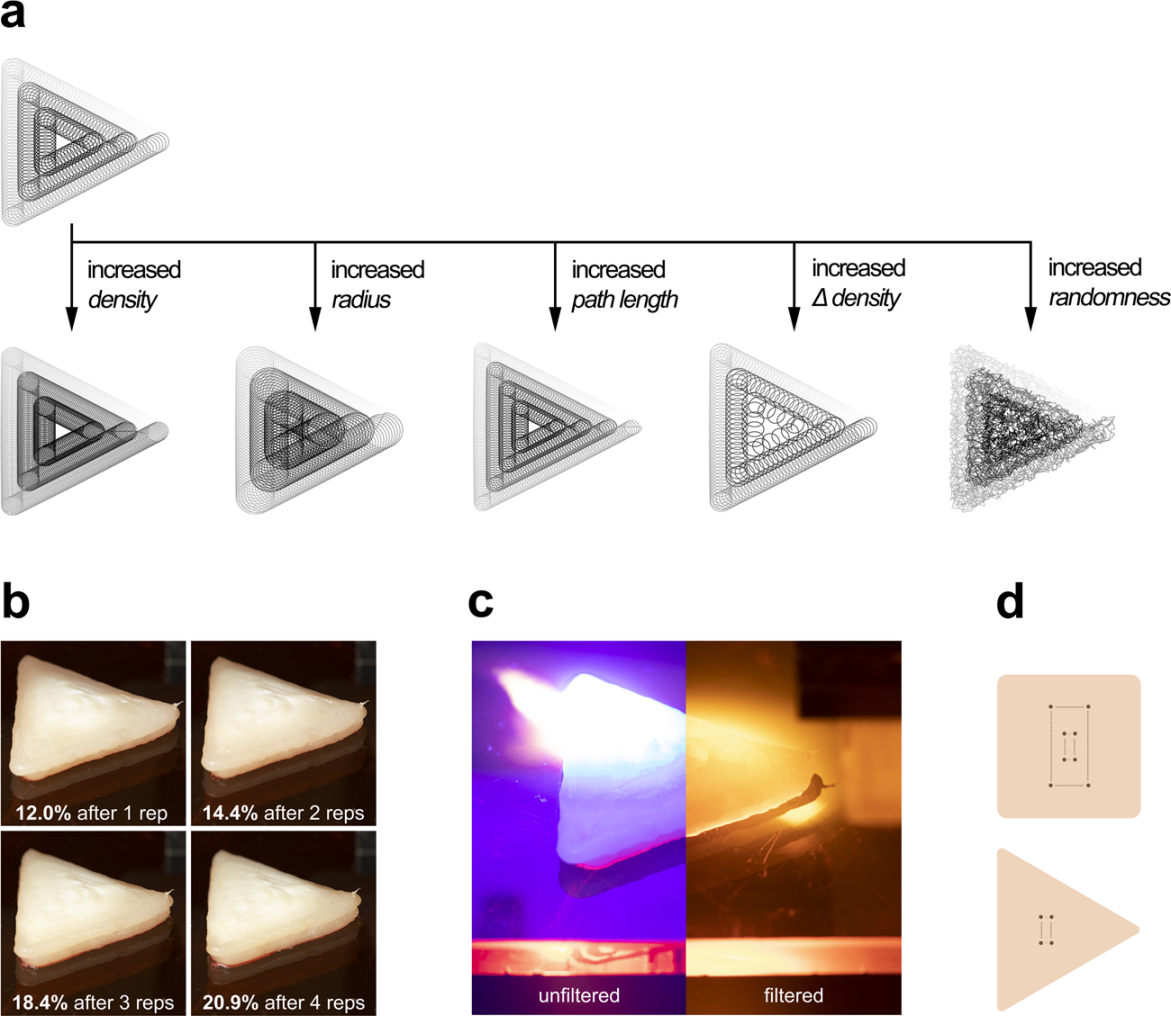
Laser cooking pattern and temperature sensing. **a** Foundational laser cooking pattern used during testing showing variations that were made to alter cooking results. Illustration is in the form of a triangle, but the perimeter can take the shape of any closed contour. **b** Shrinkage (%) of two-layer printed chicken sample documented after each repetition of the cooking pattern with the blue laser. **c** Unfiltered and filtered images of chicken being laser-cooked. **d** Eight thermocouples were placed for square prints (top) and four thermocouples were used for triangular prints (bottom).

We performed a series of trials varying laser cooking pattern (increasing density, radius, path length, Δ density and randomness) (Fig. 2a) to characterize energy requirements for laser cooking, ambient cooling effects, cooking pattern efficiency, cooking loss in heated samples (Fig. 2b), color change, and browning capabilities through packaging. Unless otherwise noted, all tests were performed on a single layer of printed chicken (~1.5 mm) (Fig. 2c).

### Energy required for food safe temperatures

**Fig. 3:**
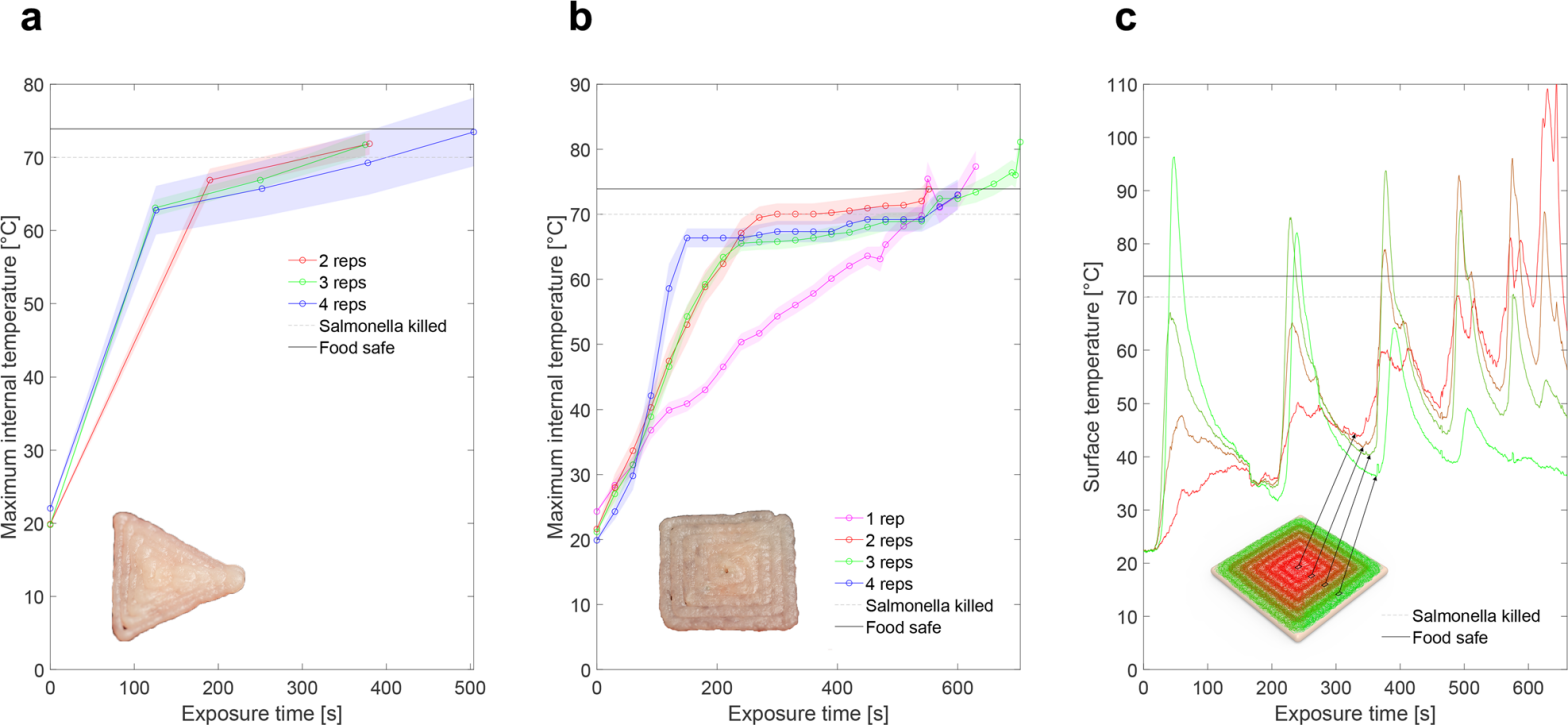
Temperature progression during cooking (each line represents a single test). **a** Maximum internal temperature achieved for triangular printed samples at various repetitions (shaded region for each line shows standard error from four thermocouples of a single sample). **b** Maximum internal temperature achieved for square printed samples at various repetitions (shaded region for each line shows standard error from four thermocouples of a single sample). **c** Surface temperature of four different voxels of chicken on the same heat-affected sample for a single rep cooking cycle. The curves range in color from green (voxel along the edge of sample) to red (voxel in the center), as does the graphic of the cooking pattern used which starts along the edge (green) and spirals in toward the center (red) of the sample.

Achieving food safe temperatures^25,26^ is vital for qualifying lasers as a processing technique. To assess thermal energy required for cooking, we traced a spiraling trochoidal path (Fig. 2a) with the blue light over the sample. More passes of the cooking pattern at higher laser speed resulted in much quicker initial temperature increases followed by much slower successive heat increases until food safe temperatures were achieved (Fig. 3a, b). Conversely, exposing the sample to a single pass of the laser at a much slower cycle speed decreased the rate at which the maximum temperature in the sample increased.

Triangular prints required 8 min while square prints required 9–10 min of consistent exposure from the blue laser to achieve an acceptable final cook temperature of 74 °C. Total laser energy required to cook a printed triangle was ~2.4 kJ and 2.7–3 kJ for a printed square (blue laser operates at 5 W). Energy requirements to reach a temperature of 70 °C^26^ and fully eradicate Salmonella—one of the more common food borne illnesses—are ~0.3 kJ less than what is required to reach a food safe temperature. Based on these energy requirements and the relative size of the two printed shapes, an energy per area of 5–7 MJ/m^2^ of printed chicken is required to achieve food safe cooking temperatures. A more powerful laser at similar energy flux would accelerate the cook time per unit area of food.

### Rate of cooling

**Fig. 4:**
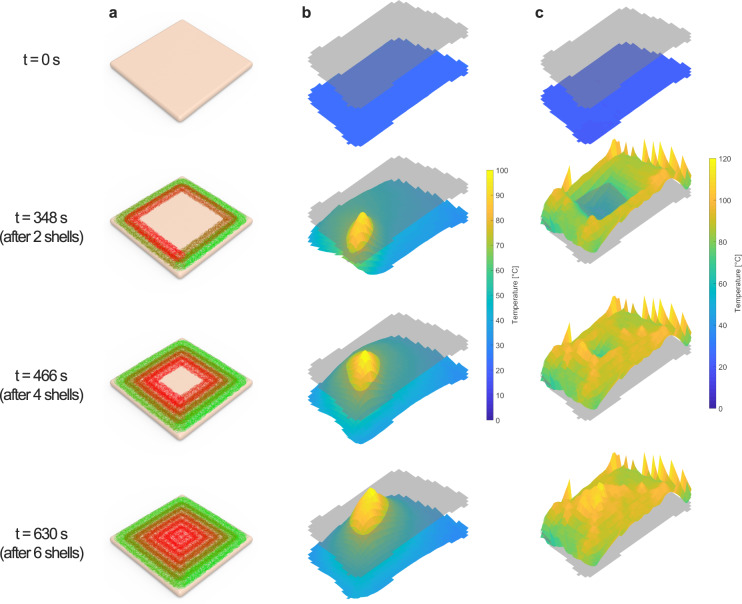
Surface temperature during laser cooking at different time stamps, t(see Supplementary Video 2 for animations of these surface plots). **a** Visual rendering of chicken sample with superimposed spiral cooking pattern showing six shells of a repeated trochoidal pattern. Pattern transitions from green (starting position) to red (ending position) throughout the duration of the time stamp to the left of each tile. **b** Infrared camera readings from surface of chicken sample at a particular snapshot in time. **c** Aggregated maximum temperature recorded on the surface of the chicken sample up until the particular snapshot in time. The 2D gray transparent plane in (**b** and **c**) shows the minimum food safe temperature needed to eradicate Salmonella and E. coli (70 °C). An IR camera was used to capture surface temperature data for the laser-heated poultry samples.

The “pulsed heating” effect from laser scanning^4^ is captured in Fig. 3c and Fig. 4 where each spike occurs when the optical center of the laser passes over a volumetric pixel—or “voxel”—of the printed sample. Because the laser scan gradually spirals toward the center of the sample, voxels along the edge of the sample have higher initial peaks and gradually lose amplitude, while voxels closer to the center of the sample have higher amplitudes at the end of the cooking cycle.

In between each pulse of laser heat, Newton’s law of cooling^27^ can be visualized (visible in Fig. 3c). The rate of cooling is dictated by the inverse response time *k* (in units of s^−1^), which determines the exponential rate by which a material cools over time. The average *k* for a voxel of chicken was ~0.0229 s^−1^ with a standard error of 0.0006. This value was computed by taking the average cooling rate across a 30 s span just after a laser pulse for nine different trials. Therefore, under normal ambient conditions, we can approximate the cooling rate as

1 $$T\left( {{{\mathrm{t}}}} \right) = T_{{{\mathrm{a}}}} + \left( {T_0-T_{{{\mathrm{a}}}}} \right)e^{ - .0229\,t}$$

where *T*(*t*) is the temperature at time *t*, *T*_*a*_ is the ambient temperature, and *T*_0_ is the temperature at time *t* = 0 s. Based on the thermal properties of chicken^28^ and the length scale of the heated sample we are assuming a Biot number that is <0.1. The rate at which food cools plays an important role in determining the population of microbial pathogens in food^29^. Therefore, by calculating the cooling rate we can more accurately predict the fluctuations in chicken temperature over time and fine tune laser parameters to ensure the food stays above a certain threshold for food safety and consumption.

### Cooking efficiency

**Fig. 5:**
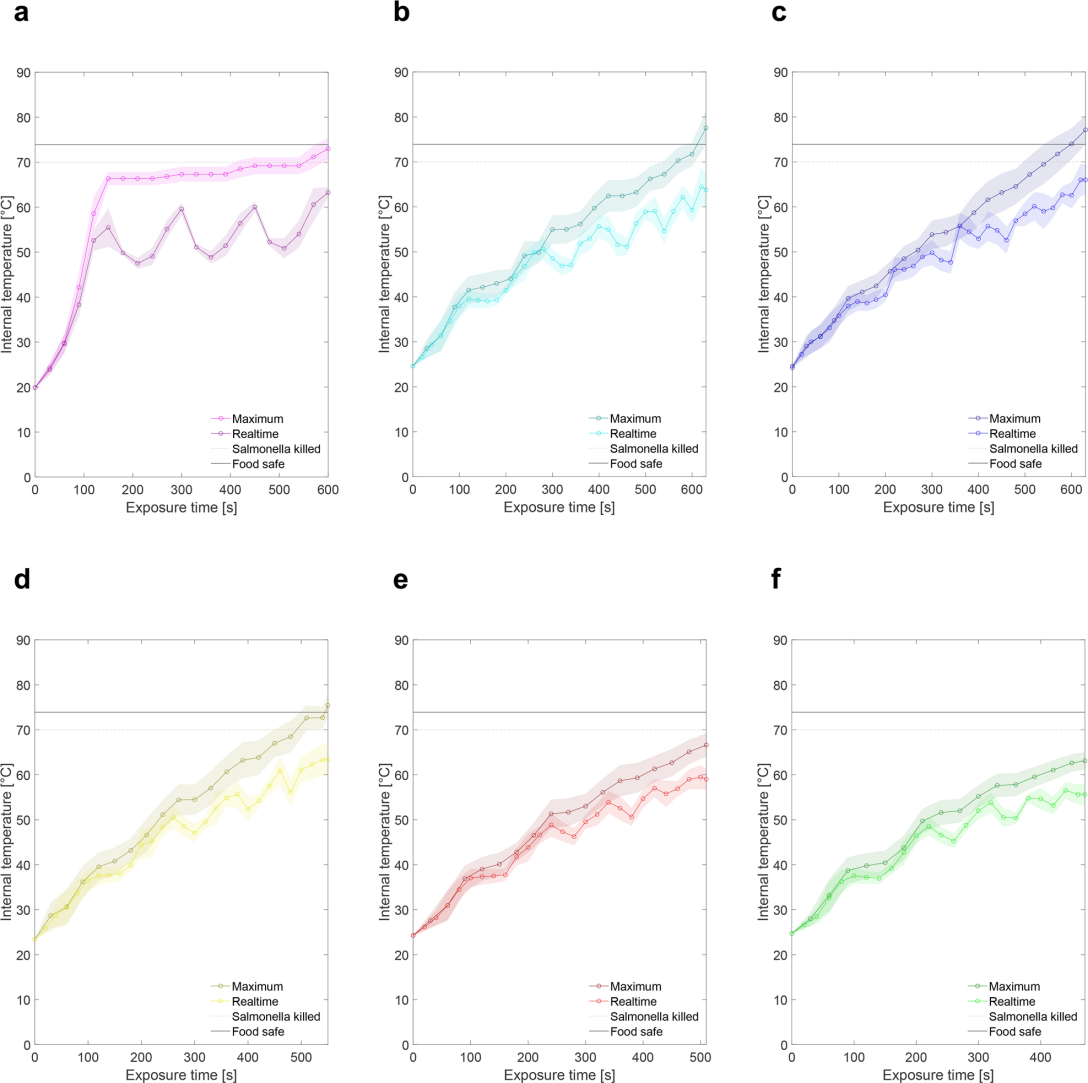
Comparing fluctuations in realtime internal temperature readings to maximum recorded internal temperature readings in laser-cooked printed chicken samples. Since the laser provides pulsed heating, regions of the food will cool unless they have direct beam exposure, which is why we are comparing realtime with maximum recorded temperature as a way of visualizing the ambient cooling taking place during the cook cycle. For the following test cases, a single printed layer of chicken was used (2.5 mm × 24 mm × 24 mm), a trochoidal pattern with six shells was used with a consistent circle radius (1.5 mm). The shape of the cooking pattern used for (**c**–**f**) was the same with the only variation being the speed of the moving laser. The density of the circles for each successive ring decreased by 0.5 rev/mm for (**c**) while remaining constant at 12 rev/mm for (**b**). Shaded region for each line shows standard error from eight thermocouples in a single sample. **a** Cycle time of 149 s (2:29) with four repetitions (9:56). **b** Cycle time of 630 s (10:30). **c** Cycle time of 630 s (10:30). **d** Cycle time of 550 s (9:10). **e** Cycle time of 511 s (8:31). **f** Cycle time of 471 s (7:51). Shaded region for each line shows standard error from eight thermocouples.

Larger discrepancies between real-time and maximum recorded temperature at various time steps (Fig. 5) correspond to less efficient heating patterns since the printed sample experiences greater heat loss to the ambient. Keeping total cook time constant, more ambient cooling is observed with multiple shorter passes (Fig. 5a) versus a single pass of the laser (Fig. 5b–f). With the blue laser pumping continuous energy into the system, maximum temperature continues to increase while real-time temperature follows an increasing oscillatory trajectory where the number of local maximum (peaks) corresponds to the number of cycles (for Fig. 5a) or shells of the trochoidal pattern (for Fig. 5b–f).

Contrary to conventional cooking on a range or in a microwave, which provides more uniform heating^30–33^, samples that are laser-cooked experience bursts of energy from the moving beam of light. As the laser propagates along the surface of the printed chicken (Fig. 4), a thermal peak follows the path of the beam as it makes contact with the food surface—also evident from Fig. 3c. Factors such as circle density, circle diameter, and speed of the laser all affect the shape and amplitude of the thermal peak.

### Cooking loss and color

**Fig. 6:**
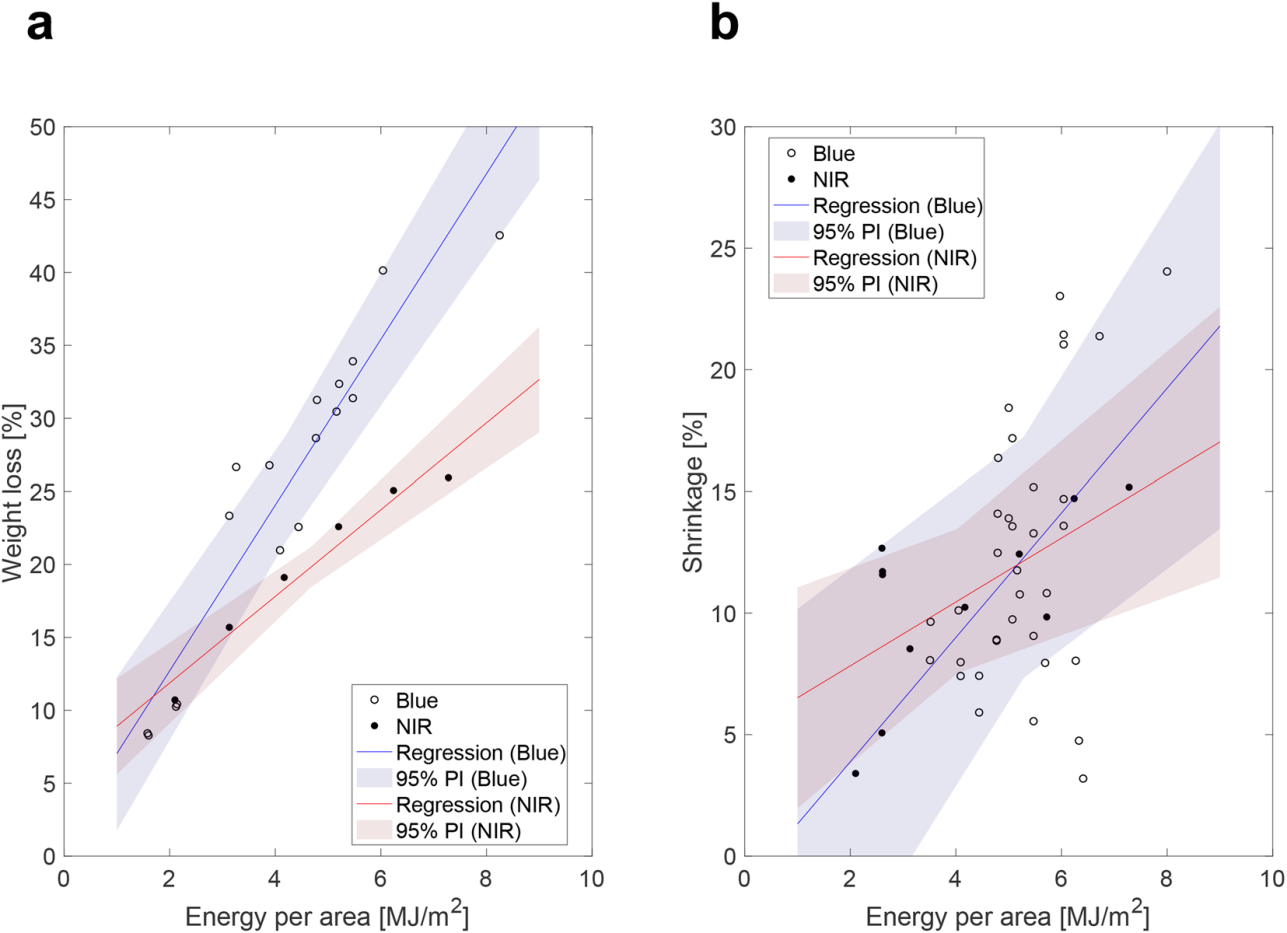
Effect of heat exposure time on weight loss and shrinkage in printed chicken samples. Each data point from each graph was from a different cooking test. **a** Weight loss percentage as a function of energy per area for laser-broiled and oven-broiled samples (sample size was 17 for blue laser tests and 6 for NIR laser tests). The power of both the blue and NIR lasers was 5 W for these trials. **b** Shrinkage as a function of energy per area for laser-broiled samples (sample size was 35 for blue laser tests and 11 for NIR laser tests). The power of both the blue and NIR lasers was varied from 5 to 10 W for these trials. Energy per area was calculated by taking the product of the laser power and the exposure time and dividing by the area of the food sample. Shaded regions represent the standard error estimate of the sample set, showing a 95% prediction interval based on the collected data.

Weight loss and shrinkage of heated poultry samples are both directly correlated to thermal exposure time (Fig. 6). Dry air heating in an oven at 300 °F (149 °C) and 400 °F^34,35^ (204 °C) was used as a comparative study. Oven-broiled samples lost nearly twice as much weight and volume as compared to laser-heated samples for comparable exposure times (Fig. 6a). Shrinkage for laser-broiled samples plateaued around 24% (Fig. 6b) while oven-broiled samples were higher around 40%. Chicken cooked via blue laser showed an increase in *L* from 70.9 to 89.6, a decrease in *a* from 7.8 to −0.5, and a decrease in *b* from 16.3 to 11.2. *L* increased in oven-broiled samples from 51.3 to 61.1, *a* decreased from 3.3 to 1.6, and *b* increased from 16.4 to 22.9. Moreover, cooking the samples with a blue laser did not result in browning on the surface of the chicken—a major reason for them having higher lightness values—while oven-broiled samples showed signs of initial browning on the edges of the crusted samples.

### Multiwavelength cooking

**Fig. 7:**
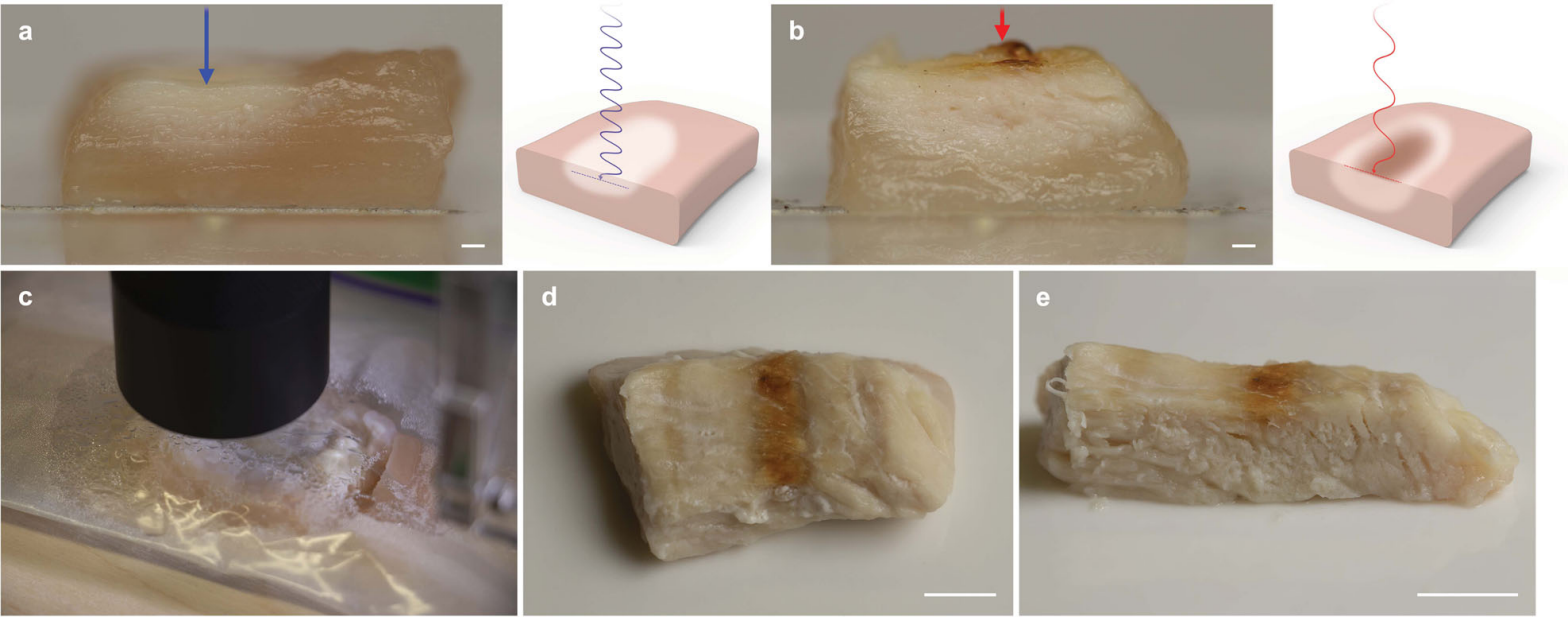
Multiwavelength laser cooking. **a** Cross-sectional cut of laser-exposed chicken from a 5 W blue laser (blue arrow) with graphic showing transmission of blue laser light. **b** Cross-sectional cut of laser-exposed chicken from an 8 W CO_2_ infrared laser (red arrow) with graphic showing absorption of IR light at sample surface. Due to the discrepancy in laser power, the 5 W blue laser performed two passes of the same trochoidal path (total exposure time = 100 s) and the 8 W IR laser was exposed to the sample for one pass with a cycle time of 63 s to ensure total energy was constant (500 J). **c** Thinly cut piece of chicken being laser-cooked by a 9 W near-infrared laser (black cylinder) within a sealed package. **d** Top view of final-cooked piece of chicken with laser-induced “grill” mark. **e** Cross-section cut of same sample. Scale bars, (**a**, **b**) 1 mm and (**d**, **e**) 1 cm.

Blue light achieved subsurface cooking to a depth of 2.4–3 mm (Fig. 7a) with no immediate browning to the surface. Samples heated via IR laser (Supplementary Fig. S2, right) had an HAZ nearing 3.7–5 mm deep (Fig. 7b). Variations in cooking depth can be attributed to differences in sample thickness (5–6 mm); thicker samples dissipate more thermal energy to the surrounding raw meat. While the heat-affected zone (HAZ) of the blue laser was smaller, heat from the blue light penetrated deeper into the sample. Conversely, IR light was immediately absorbed by the food and resulted in browning. The larger HAZ, from the sample exposed to the IR laser, can be attributed to heat conduction and higher laser power (IR laser was operating at 8 W and the blue laser was operating at 5 W). Due to the high heat absorption by water of IR light, the beam needed substantial defocusing to prevent the sample from vaporizing (IR laser intensity ~5 W/cm^2^, blue laser intensity ~60 W/cm^2^). Three samples were used to assess cooking depth for the blue laser and six samples were used for the samples cooked via IR laser (Supplementary Fig. S3).

### Cooking through packaging

Exposing raw poultry samples—sealed in plastic packaging—to blue and near-infrared (NIR) light resulted in color changes consistent with protein denaturation^36,37^ with no surface browning (Supplementary Fig. S4, left). Enclosing the sample reduced moisture evaporation and browning development by limiting oxygen exposure^20^ (visible in Supplementary Fig. S4, right). Using a trochoidal heating pattern with the NIR laser and leaving an air gap between the plastic enclosure and the top surface of the sample (Fig. 7c), we achieved a brown crusted region along the food surface (Fig. 7d, e) resembling a “grill” mark.

Slight browning was achieved on the surface of the sealed chicken sample with the blue laser. Moisture evaporation from the heated food beaded into water droplets along the inside of the bag (Fig. 7c). While both lasers induced protein denaturation (Fig. 7d and Supplementary Fig. S4, left), the NIR laser was more efficient for browning foods through packaging. Slower cooking speeds (~50 mm/min) encouraged heat build up and were most effective for browning with the NIR diode laser.

### Spatial resolution of laser cooking

**Fig. 8:**
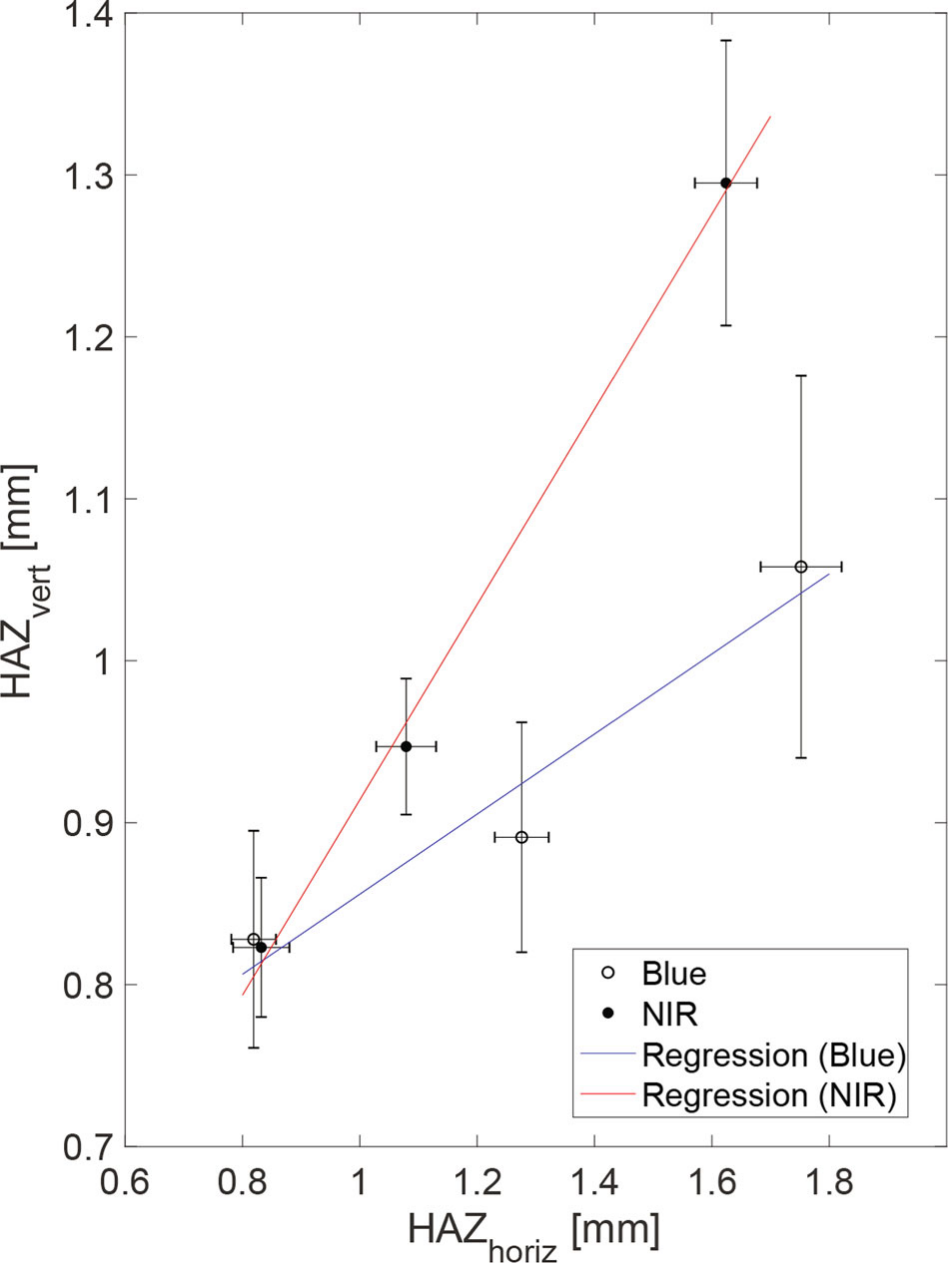
Comparing horizontal and vertical heat-affected zones (HAZ) from laser cooking. Maximum depth is plotted against maximum width of the HAZ. Each data point presents a standard error that was tabulated from five repeated experiments (*n* = 5). The values from each experiment were measured three times and averaged to minimize the error associated with the measuring technique. Information regarding the method used to calculate HAZ can be visualized in Supplementary Fig. S17. Error bars represent standard error of the mean.

We propose a laser-based cooking approach and calculate the cooking resolution to be ~1 mm. To quantify this precision, we exposed a 0.25 in (6.4 mm) thick sample of chicken to a blue laser (Supplementary Fig. S5), an NIR laser (Supplementary Fig. S6), and an MIR laser (Supplementary Fig. S7). Lines were etched onto the surface of the food and cross-sectional cuts were made to assess the HAZ. Figure 8 shows the resulting linear relationship between the horizontal and vertical depth of the HAZ for the blue and NIR lasers. The MIR laser operated at a power and speed that only resulted in surface cooking with no penetrative heating. The relationship between horizontal (*W*_laser_) and vertical (*D*_laser_) HAZ followed

2 $$D_{{{{\mathrm{blue}}}}} = 0.247 \times W_{{{{\mathrm{blue}}}}} + 0.609$$

3 $$D_{{{{\mathrm{nir}}}}} = 0.603 \times W_{{{{\mathrm{nir}}}}} + 0.311$$

for the blue (Eq. 2) and NIR (Eq. 3) lasers, respectively.

## Discussion

Software-controlled laser cooking offers a higher degree of spatial and temporal control for food processing than conventional cooking methods^4,38^. Lasers can provide broiling, browning, or penetrative cooking by varying the laser beam wavelength and scanning pattern. Near- to mid-infrared lasers can provide surface level browning and broiling, while blue lasers are more tailored toward penetrative cooking. Here, we used a 5–10 W laser for diffuse heating, but our laser cooking apparatus can seamlessly integrate with a higher powered visible or infrared laser, allowing the beam to move faster over the food sample since energy flux is greater, thereby decreasing total cooking time. Increasing laser power might contribute to more penetrative cooking since more energy is distributed across the surface of the food, allowing for more heat to be conducted through the thickness of the food sample. Decreasing cooking times and achieving higher heating penetration would enable this technology to be utilized in commercial cooking applications.

We found the wavelength and scanning pattern of the laser are key heating parameters that affect final cook quality. Due to the shallow penetration depths of this radiative heating method, digitally-controlled laser cooking is more suited to thin-layered food products and in situ cooking on a 3D food printer: the layer thickness of which closely resembles the heating penetration of a blue laser. Laser cooking can function independently to cook thin-layered food, could be integrated into commercial food printers^39^ or more traditional kitchen heating appliances to provide tunable cooking and customizable aesthetic expression on cooked food^40^.

Millimeter-scale precision allows printing and cooking a burger that has a level of doneness varying from rare to well-done in a lace, checkerboard, gradient or other custom pattern. Heat from a laser can also cook and brown foods within a sealed package. Cooking foods that are hermetically sealed could significantly increase their shelf life by reducing their microbial contamination^41^ and has great commercial applications for packaged to-go meals at the grocery store, for example. We also had two blind taste-testers sample laser-cooked and range-cooked printed chicken and they both seemed to prefer the laser-cooked sample due to the fact that it remained more moist and the texture throughout was more uniform (see Supplementary Materials for more information).

While this study only uses chicken as a model food system, laser heating of grain-based substrates—that more readily absorb water—should accelerate moisture loss and browning during cooking as well. Future studies could also investigate (1) the effects of heating on layer adhesion between successive cooked printed layers, (2) simultaneous multiwavelength cooking for both penetrative and surface heating, (3) methods to reduce cross-contamination between cooked and raw printed layers, and (4) the effects of food cooling rates on lethality to further understand commercialization potential. Software cooking is a relatively uncharted space^2^ and multiwavelength cooking boasts interesting opportunities for tailored meal creation and can be extended to other animal proteins^4,23^ or food groups.

## Methods

### Sample preparation

Raw chicken breast was purchased from a local convenience store (Appletree Market, New York, USA) and blended in a food processor for a few minutes (Cuisinart FP-8GMP1, Stamford, USA) until a uniform consistency was achieved. To avoid clogs during deposition, tendons were removed and samples were refrigerated and repacked into syringe barrels each day. Each syringe barrel (PN: 7012134) was outfitted with a 14 gauge tapered nozzle tip (PN: 7018052) (Nordson FD, East Providence, USA).

### Printing food

Due to the material’s viscoelastic properties, syringes needed to be primed—similar to what conventional filament-based plastic 3D-printers do—and expel material prior to printing the edible constructs. The starting height (*z*-height) of the nozzle needed to be calibrated prior to each print. An effective *z*-height is when the layer thickness is slightly higher than the distance between the nozzle and the printing surface. Because we used a tapered 14 gauge syringe tip (1.5 mm internal diameter), an effective starting vertical height was ~1.2 mm (~80% of nozzle outlet diameter) above the print bed. A custom acrylic plate was used as a build plate to (1) absorb stray blue and NIR laser light and (2) fixture the thermocouples for data collection. Lastly, we used a feed rate of 600 mm/min, which provided controllable and repeatable deposition paths.

### Cooking apparatus

Our selective laser broiling apparatus consists of five main parts: (1) a custom 3D-printed fixture, (2) a high-powered diode laser, (3) a set of mirror galvanometers (a.k.a. “galvo mirrors”), (4) laser shielding, and (5) a removable tray for material placement. The printed fixture orients the blue diode laser toward the dual-axis galvo mirrors and can accommodate a modular laser rack (Supplementary Fig. S2, left)—analogous to an oven rack—to vary working area and intensity of the beam.

During initial laser cooking trials, our laser diode was mounted in the 3D-printed fixture (described above), but as the experiments progressed we transitioned to a setup where the laser was vertically mounted to the head of the extrusion mechanism. This setup allowed us to print and cook ingredients on the same machine. We designed and fabricated an acrylic platform and printed fixture that allowed us to seamlessly mount the laser via the slotted holes in its base (Supplementary Fig. S2, right). Laser diode calibrations were conducted with a Newmark thermopile sensor (PN: 919P-150-26) (MKS Instruments, Inc., Irvine, USA). A linear relation was observed between the current and the power output of the blue and NIR lasers and was found to be

4 $$P_{{{{\mathrm{blue}}}}} = 9.2774 \times I_{{{{\mathrm{blue}}}}} - 2.9104{{{\mathrm{,}}}}\;{{{\mathrm{R}}}}^2 = 0.9994$$

5 $$P_{{{{\mathrm{nir}}}}} = 0.9888 \times {\it{I}}_{{{{\mathrm{nir}}}}} - 0.2205,\;{{{\mathrm{R}}}}^2 = 0.9979,$$

respectively.

Control cooking experiments were performed in a toaster oven (Breville BOV800CRNXL) (Sydney, Australia) at 400 °F (204 °C), which is based on common cooking temperatures for oven-broiled chicken^34,35^. Typical cooking times for boneless chicken breast in the oven is ~25 – 40 min for four fillets (~1 lb)^42^ and since our printed samples are only ~45 g each, we cooked each printed sample for 3 min.

### Controlling the mirror galvanometers

We used a Raspberry Pi 2 Model B (Raspberry Pi Foundation, Cambridge, United Kingdom) to control our dual-axis mirror galvanometers (Seeed Studio Electronics, Shenzhen, China). The digital output signal of the microcontroller (0–5 V) was converted into a 12-bit analog signal via the use of digital-to-analog converters (MCP4725, Adafruit Industries, New York, USA) and the voltage range was extended to 24 V to allow for full range of motion for the galvo mirrors. A custom fabricated PCB (Supplementary Fig. S8) amplified the signal to our desired 24 V range. Each signal controlled a galvo and gave use directional control of the beam (Supplementary Fig. S9). We then created cooking patterns in MATLAB^®^ and converted them to actionable motor commands.

### Image acquisition and processing

All pictures were captured with a digital single-lens reflex camera (EOS 80D, Canon, Tokyo, Japan) using an EF 100 mm macro lens (Canon, Tokyo, Japan). Consistent lighting was ensured through the use of a white light box and an aerial light positioned overtop of the samples. We shot with a color swatch for white balancing, which was the only post-processing performed on the photos in Adobe Photoshop CC.

### Food printer design

We retrofitted a commercially available X-Carve (Inventables, Inc., Chicago, USA), an off-the-shelf three-axis Cartesian gantry, with a custom extrusion mechanism (Supplementary Figs. S10a, S11). The extruder and all axes of the gantry were driven by ClearPath brushless servomotors (CPM-SDSK-2311S-RQN) (Teknic, Inc., Victor, USA) and controlled by a Smoothieboard, an open-source controller used to drive traditional 3D printers and CNC machines. Our extruder accommodates a 30 mL syringe barrel (PN: 7012134), a 14-gauge flexible tapered nozzle tip (PN: 7018052). The extruder axis utilizes a motor-driven lead screw. We used Repetier-Host (Hot-world GmbH & Co. KG, Willich, Germany), a free 3D printing application, as a front-end interface to maneuver and run cooking programs, which were written in G-code, a common numerical language used to control machinery.

### Generating print and cook files

We developed a custom MATLAB^®^ script giving us control of an object’s maximum width, shape, and height and convert this into a file format for printing and cooking. The script allows the user to control extruder flow rate and print speed as well and outputs a G-code file that controls food deposition and a text file to control each galvo mirror. A program on the Raspberry Pi sequentially reads the text file, converting them to motor commands, and generates produces a cooking pattern.

### Laser cooking pattern design

Based on prior research^4,20^, the lasers trace a trochoidal ring pattern that follows the deposition path of the printed layer (Fig. 2a). Because the heating pattern is parametric, variables such as density, diameter, speed, and stochasticity of the repeating rings can be optimized for more or less heating in a particular region of the food sample. To further compensate for the galvo mirrors’ slight imperfections, the pattern was stretched in the *x* direction by a factor of 1.35 and rectilinear distortion was added (Supplementary Fig. S12c). Galvo mirrors often times suffer from pincushion distortion, which is a type of optical distortion where straight lines closer to the edges of the mirrors’ range of motion bulge toward the center. To counter this, we applied a slight barrel distortion (Supplementary Fig. S12b) which allowed us to trace more true-to-shape geometries.

### Measuring temperature

K-thermocouples (±1 °C) were used to capture the internal temperature of the printed chicken samples (four sensors were used for triangular prints and eight sensors for square prints). Spherical probes were placed into slotted holes that were cut into the acrylic platform that supported printed samples, which was 1.5–3 mm thick. Surface temperatures were recorded using a FLIR C2 Compact Thermal Imaging System (±2 °C or 2%, whichever is greater) (Wilsonville, USA). This IR camera captures a 60 × 80 pixel array at 2.5 fps that we post-processed in MATLAB^®^ to create time-lapse surface plots (Supplementary Video 2).

### Data processing

MATLAB^®^ scripts (Supplementary Script 1) were generated to measure changes in (1) surface color, (2) surface temperature, (3) weight, and (4) volume (i.e., shrinkage). We used the *Lab* color space in our color analysis and performed measurement processes in triplicate for each imaged sample. Samples used for data collection can be seen in Supplementary Figs. S13 and S14.

To measure changes in surface color, sampling regions were selected manually (Supplementary Fig. S15) with the intent that regions would be selected based on low—to no—specular reflectance from the light source. We manually sampled regions for color information to avoid white-shifting the color data due to specular reflectance from surface imperfections.

Pixel data from the surface temperature recordings need to be filtered prior to creating three-dimensional surface plots (Supplementary Video 2). First, we removed any pixels that registered a temperature greater than the maximum temperature recorded on the sample; this eliminated pixels along the edge of the sample that were registering temperature peaks on the surrounding acrylic piece. Second, any pixels that logged a temperature above the starting temperature of the food sample would be eliminated.

Weight loss was recorded using an analytical balance (0.1 mg resolution) (Denver Instrument, Arvada, USA) to capture the precision weight changes in the printed food samples.

Finally, shrinkage was calculated by way of identifying food-containing pixels using color from the images that we captured; we make the assumption that the samples shrink uniformly in all directions. Our script allowed the user to manually select two points (black dots in Supplementary Fig. S16a) to trace out a region on the food sample. The script grabbed the lowest and highest RGB color values within this selected region (red-dotted square in Supplementary Fig. S16a) and kept any pixels that fell within this color range (yellow region in Supplementary Fig. S16b).

### Measuring spatial resolution of the lasers

We used the change in color of the heated chicken to determine the extents of the HAZ. A custom MATLAB^®^ script (Supplementary Script 2) was written to calculate the depth and width of the heat penetration based on the selection of a few points on the chicken samples (Supplementary Fig. S17). This process was executed three times for 30 different pictures. Five tests were performed at three different speeds for each of the lasers, and measurements were repeated three times to minimize the error associated with the measuring technique. Blutinger et al. (2019)^1^ utilized a similar method to measure the HAZ of laser-cooked salmon.

### Taste-testing

We fully cooked two separate samples of printed chicken using a blue laser and an electric range for taste-testing. Two taste-testers were given two plates: (Sample 1) printed chicken cooked using a blue laser, and (Sample 2) printed chicken cooked on an electric range. We did not tell the testers how each sample was cooked but they were aware that one sample was laser-cooked and one sample was range-cooked. Each tester was instructed to taste each sample and describe all sensory reactions. Their responses are reproduced verbatim in the Supplementary Materials file.

## Supplementary information

Supplementary Materials

Supplementary Video 1

Supplementary Video 2

Supplementary Script 1

Supplementary Script 2

## Data Availability

All data are available from the authors upon reasonable request.

## Code availability

All code used to generate supporting figures can be found in the Supplementary Information.
