# Supplementary material for: Precision cooking for printed foods via multiwavelength lasers
Source: NPJ Sci Food. 2021 Sep 1;5:24. doi: 10.1038/s41538-021-00107-1 (PMC8410778; doi:10.1038/s41538-021-00107-1)
Supplement: Supplementary file 1 — Supplementary Materials [file 41538_2021_107_MOESM1_ESM.docx]

**Supplementary Materials**

**Precision cooking for printed foods via multi-wavelength lasers**

Jonathan Blutinger^[[1]](#footnote-1),*^, Alissa Tsai1, Erika Storvick1, Gabriel Seymour1,

### Elise Liu1, Noà Samarelli1, Shravan Karthik1, Yoran Meijers1,^[[2]](#footnote-2)^, Hod Lipson1

**Contents:**

Descriptions……………………………………………………………………………............................... 2

Supplementary Data………………………………………………………………………………………… 3

Supplementary Figures…………………………………………………………………………………….. 5

**File name:** Supplementary Materials

**Description:** Supplementary Data and Supplementary Figures.

**File name:** Supplementary Video 1

**Description:** Precision laser cooking overview. This video provides a summary of the paper. We introduce laser cooking as a new digital processing method, describe the experimental method, provide an overview of the cooking apparatus, and illustrate the main results.

**File name:** Supplementary Video 2

**Description:** Temperature surface plots during laser cooking. This video shows surface temperature surface plots of various cooking trials. Maximum surface temperature and maximum internal temperature can be compared for various cooking trials that vary in exposure time and number of passes.

**File name:** Supplementary Script 1

**Description:** MATLAB script used to process data and generate graphs.

**File name:** Supplementary Script 2

**Description:** MATLAB script used to calculate depth and width of heat-affected zone.

**Supplementary Data**

Responses are reproduced verbatim in *italics* with occasional follow-up questions from the overseer of the experiment in **bold**.

Taste-tester 1

[Responses from eating Sample 2]

*It’s got a few brown spots on the top so it looks like it’s pretty cooked. It smells pretty precisely liked cooked chicken, it smells like it’s cooked. You can’t smell any like machine, straight chicken. It tastes fully cooked, the texture is conventionally like a dryer chicken. A little dry but cooked throughout. Durable, solid, no soft spots, pretty hard throughout. It’s good, if I liked cooked chicken I would eat that regularly with no seasoning.*

[Responses from eating Sample 1]

*Looks a little different. Looks a little whiter in the middle and more on the outside for browning. This one smells a little more metallic I guess. Let’s try it.. Cooked throughout, a little bit smoother on eating. A little bit less dry than the other one, surprisingly. More moist. Certainly not rubbery, it was good. The stringiness from the first one wasn’t there. Traditional raw chicken kind of breaks up in your mouth, this one didn’t break up as much, it’s more solid throughout. More uniform structure, tastes more uniform.*

[Could identify which sample was laser-cooked]

**What do you mean by metallic?**

*Ever go to the dentist and get fillings done? They have a laser they use to seal the fillings and you get that smell.. a little bit of an industry odor a sharpness you get to it that you don’t get with normal chicken. You smell the heating of the laser and you can smell it a little bit with the chicken, it leaves a little bit left on it. Sample 1 you can smell a traditional cook. Normally with cooked foods you can taste what it has been cooked in oil if or if it’s been baked. It was very slight but I like consistency more of the laser-cooked.*

**The texture is better?**

*Yes, it didn’t have as many different parts to it. The outsides of Sample 1 were a little bit crispier than the insides of the cooked chicken, and this one (Sample 2) was more uniform, there were no breakups in the consistency of the chicken.*

Taste-tester 2

[Responses from eating Sample 1]

*The taste is chicken. It tastes like chicken. The first bite is a little squishy but then as it tears apart in your mouth it feels right. It evens out pretty well. It’s not really dry. I’d eat it, it’s good chicken. My mouth didn’t dry out eating it.*

[Responses from eating Sample 2]

*This is definitely a little bit more dry. The first bite wasn’t quite as chewy as the other one (Sample 1), but it is definitely more dry. The taste, I think is pretty much equivalent. The texture is similar but this one being dryer you have to chew it a bit more so it’s harder to get through.*

**Which one did you prefer in terms of texture?**

*This one (pointing to Sample 1), it was easier to chew. Seemed more moist.*

**And that’s important?**

*Yes, especially with meat.*

**Supplementary Figures**


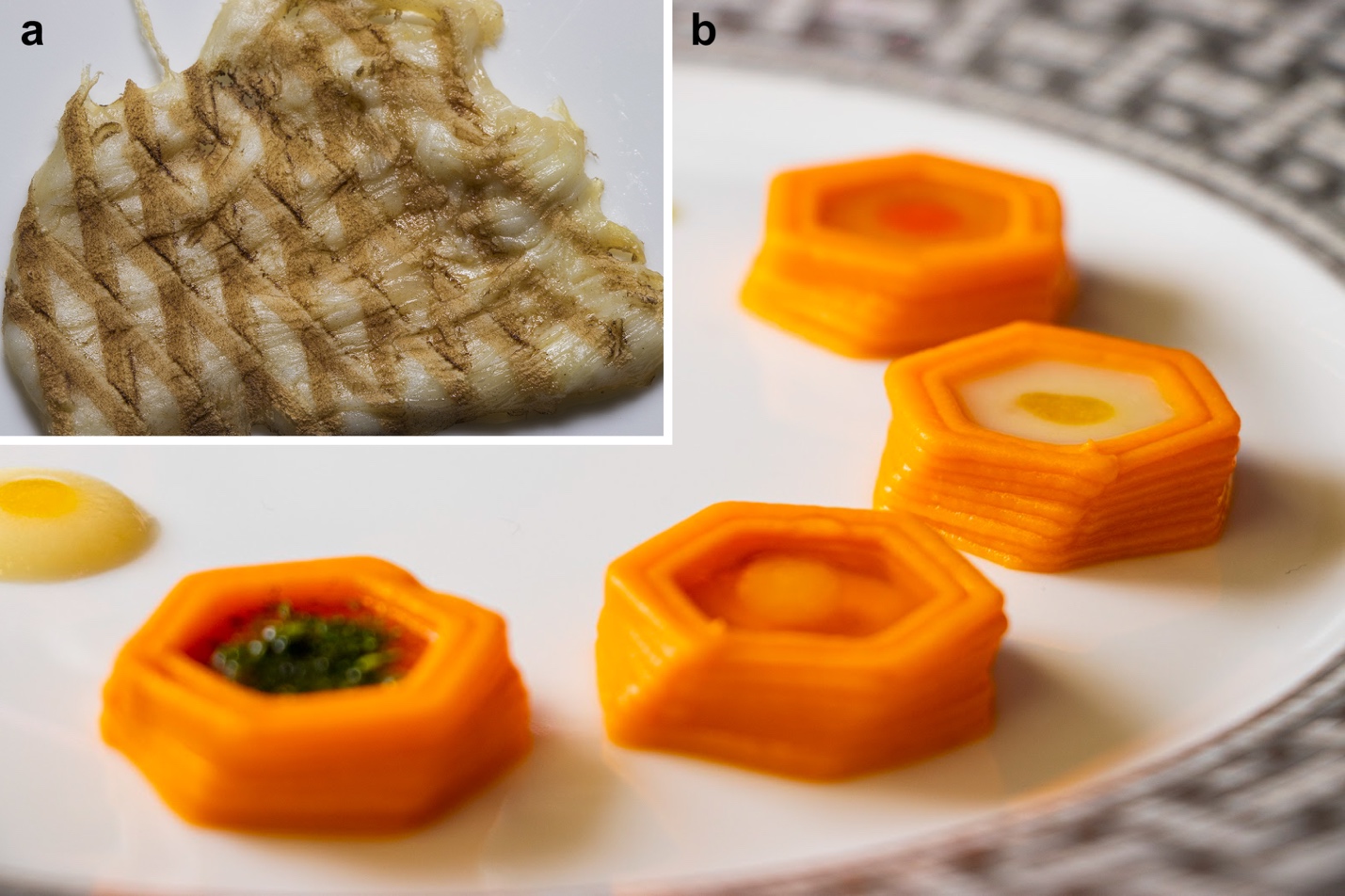


***Fig. S1:*** ***a*** *Chicken breast slice that has been cooked using a blue laser and browned using a CO_2_ infrared laser.* ***b*** *Printed hexagonal structures made from a carrot purée with various fillings.*

**
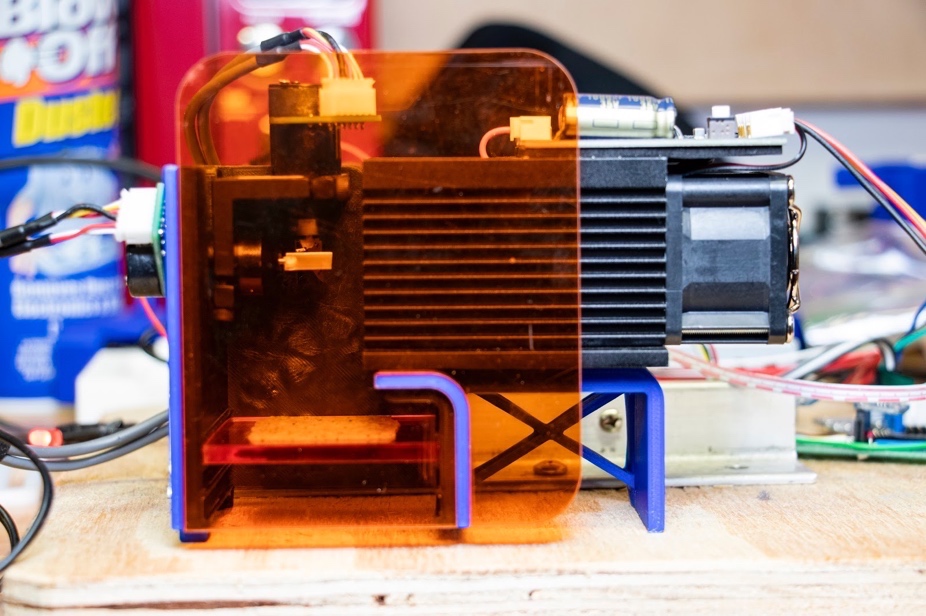
**
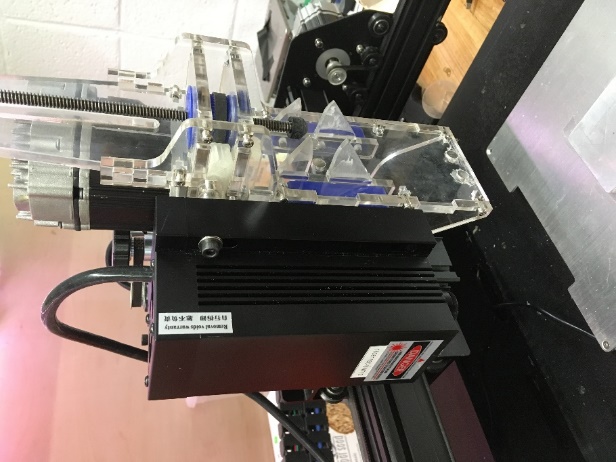


***Fig. S2:*** *Laser systems used in cooking trials.* ***Left****: Stationary blue diode laser with energy being redirected by dual axis mirror galvanometers.* ***Right****: Near-infrared diode laser mounted to a 3-axis gantry.*


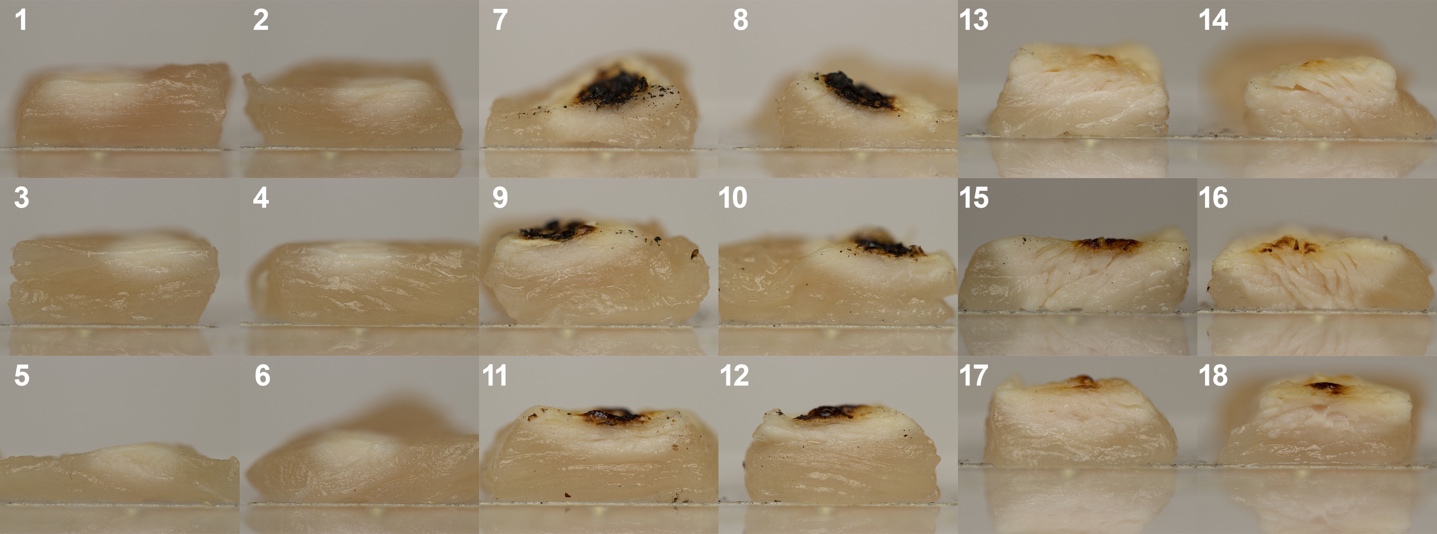


***Fig. S3:*** *Cross-sectional views of testing samples used to assess cooking depth. Samples 1 – 6 were heated with a 5 W blue laser. Samples 7 – 18 were heated with an 8 W CO_2_ IR laser.*


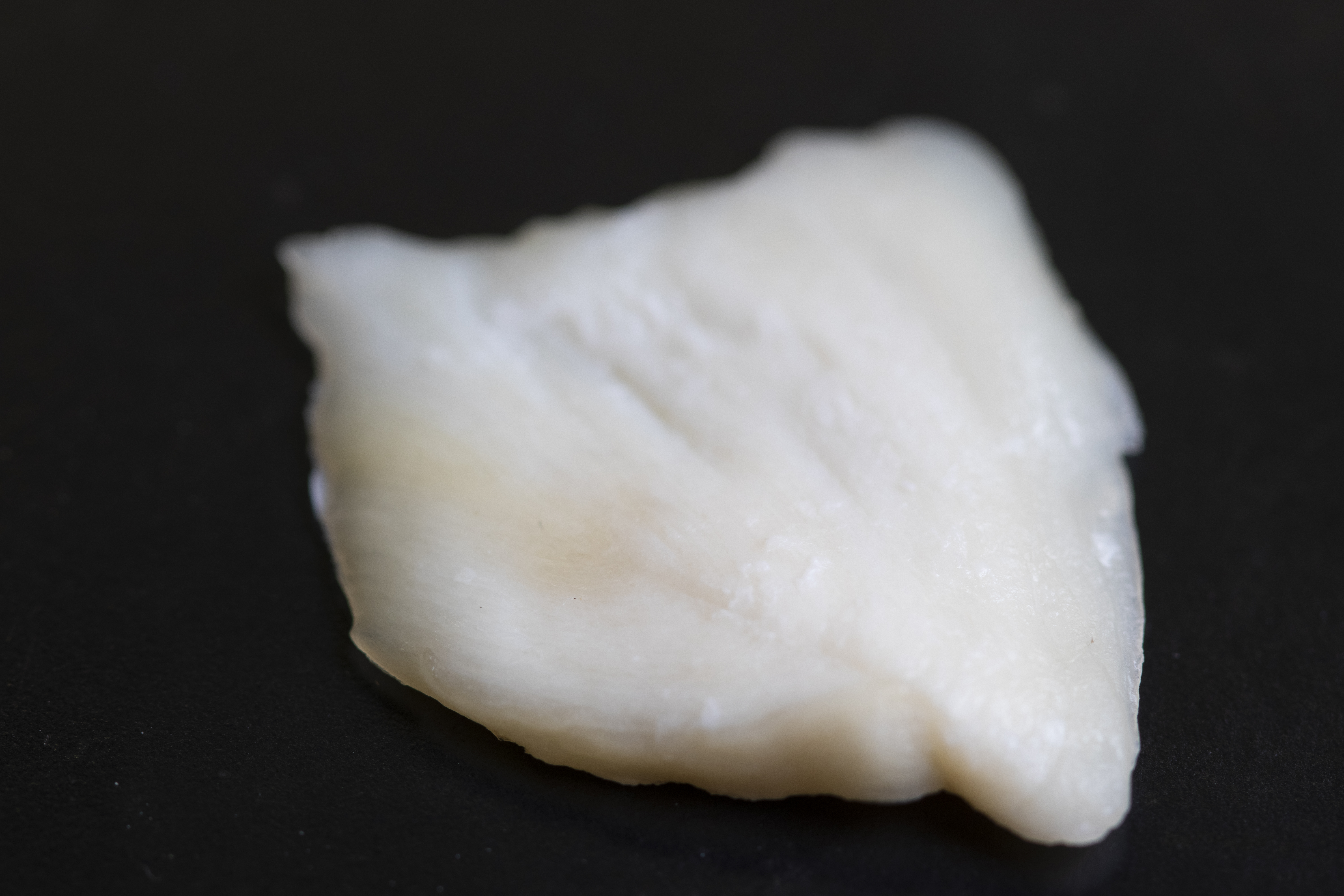

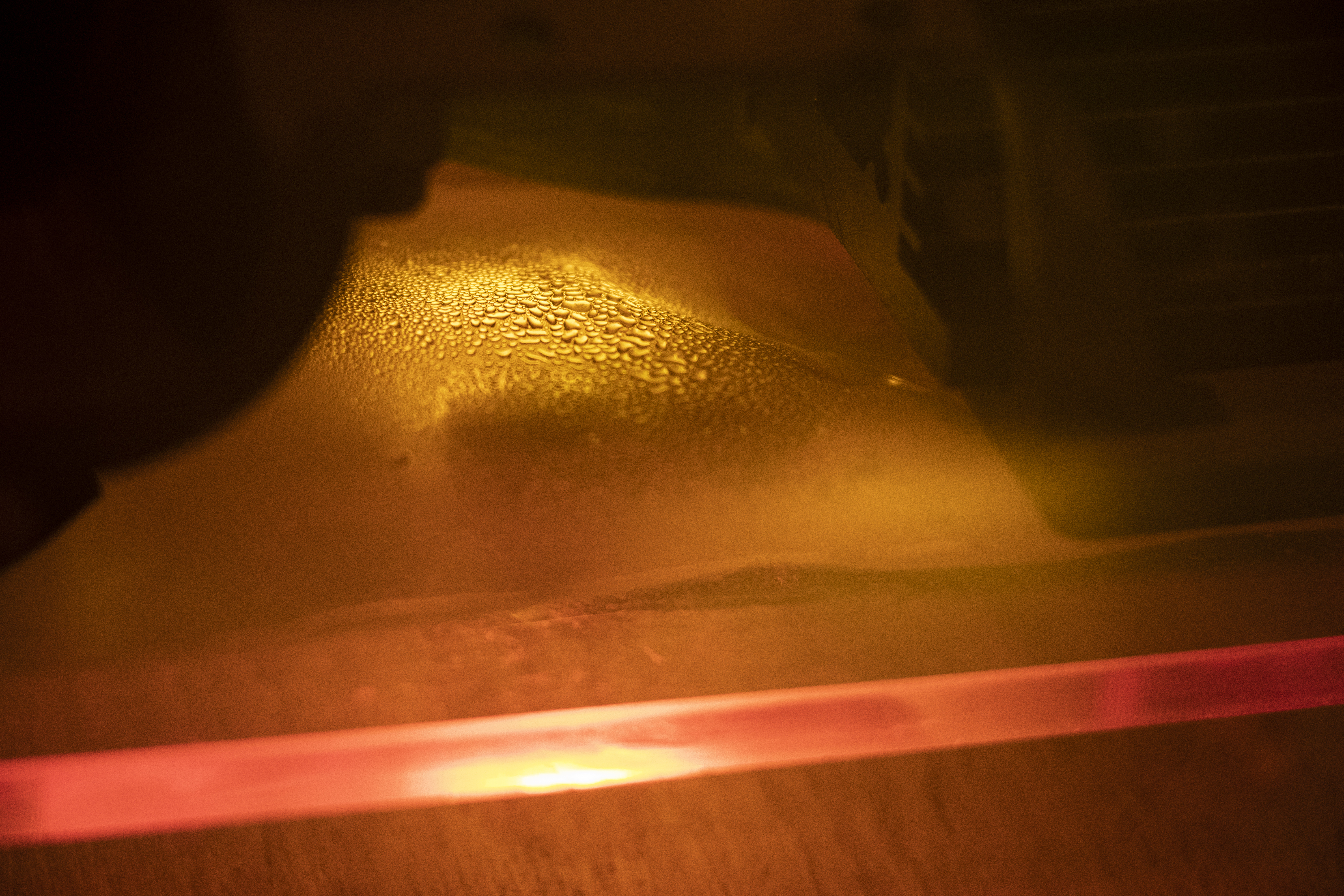


***Fig. S4:*** *Cooking chicken with a blue laser through packaging.* ***Left****: Chicken that’s been laser-cooked via blue laser showing protein denaturation with little to no indication of surface browning.* ***Right****: Chicken sealed in a plastic package being cooked by a blue laser with visible condensation on the interior of the package.*


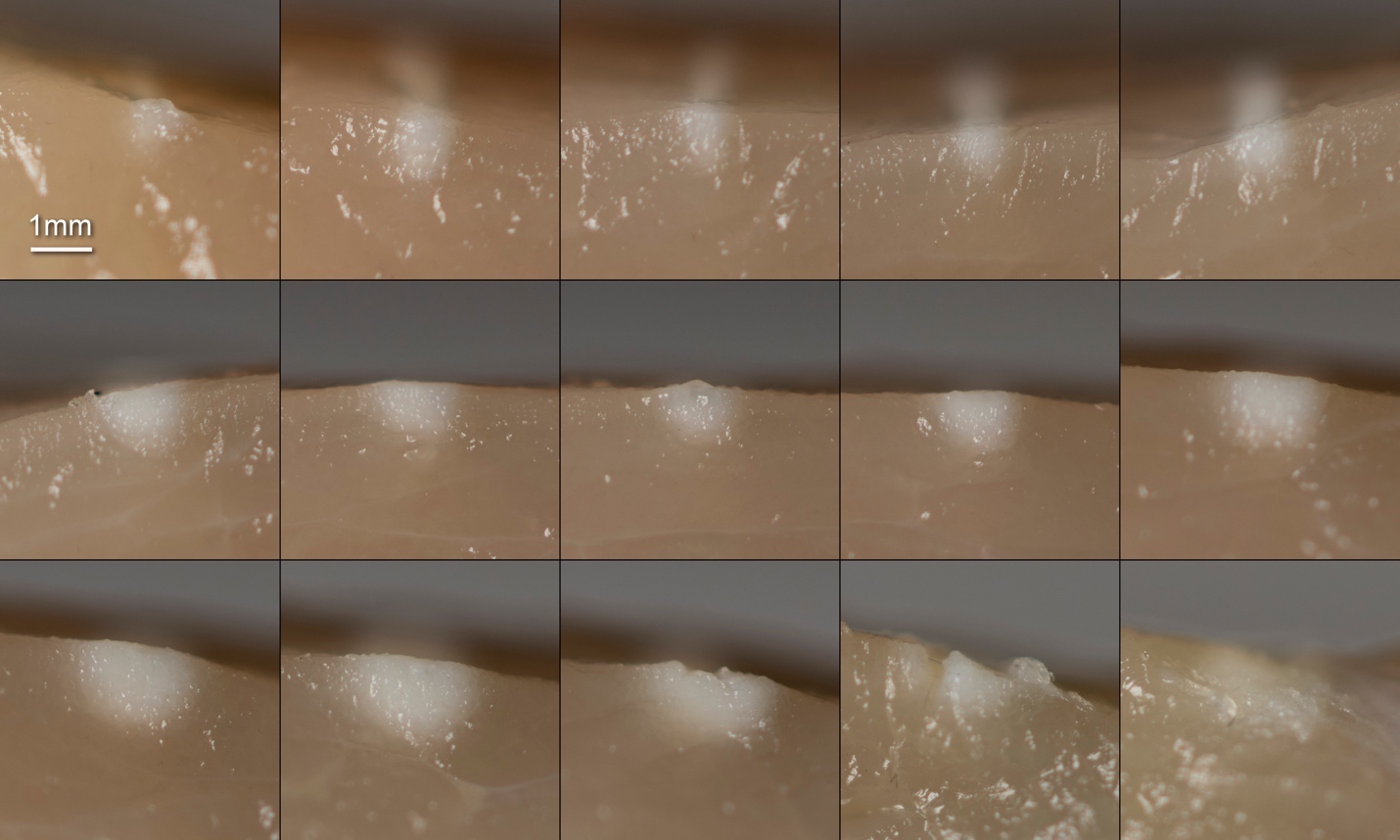


***Fig. S5:*** *Samples used to calculate horizontal and vertical heat-affected zone for chicken cooked via blue laser. Laser power was held constant at 10 W for all tests. Each row corresponds to a different cooking speed (top: 200mm/min, middle: 170 mm/min, bottom: 140 mm/min). All images are cross-sectional cuts showing a side view.*


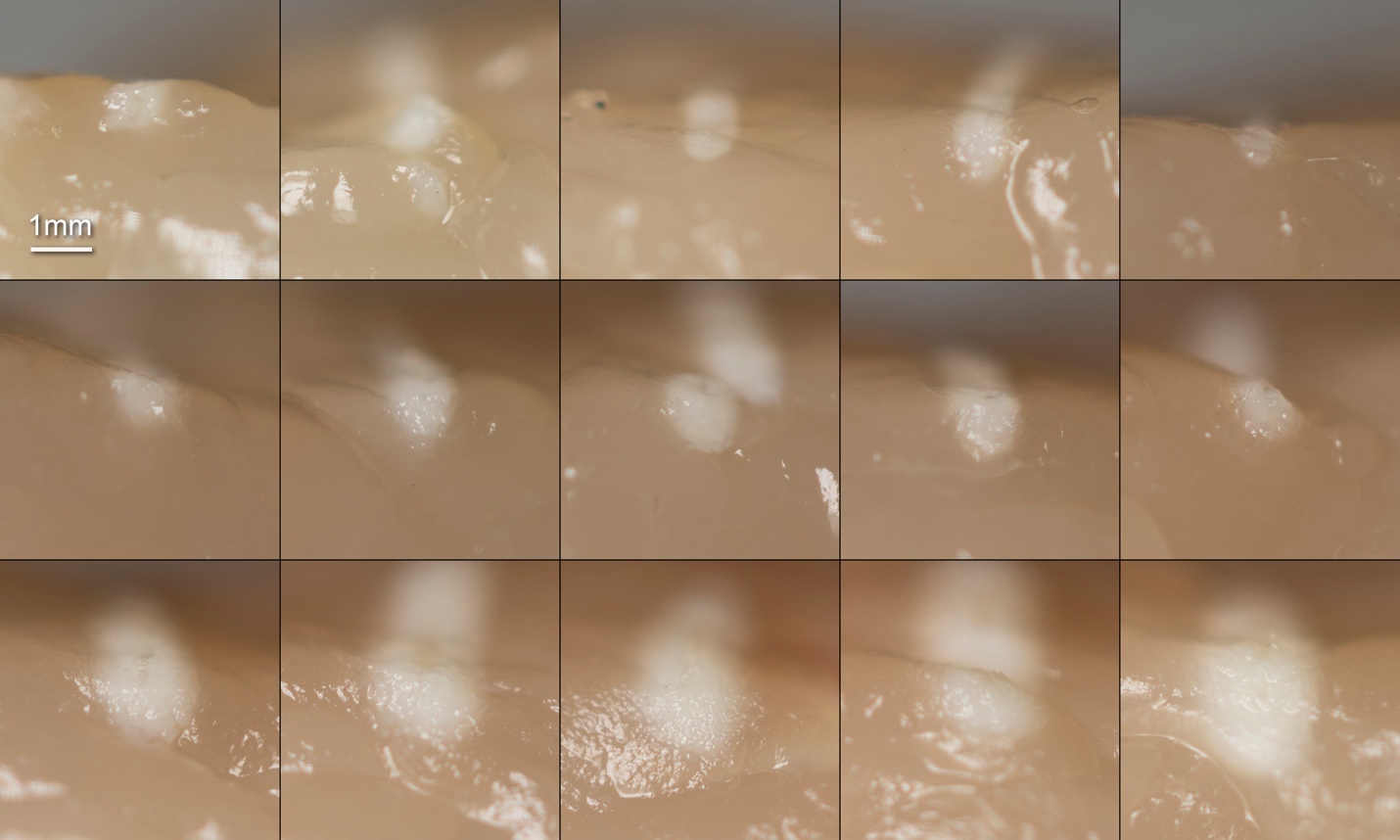


***Fig. S6:*** *Samples used to calculate horizontal and vertical heat-affected zone for chicken cooked via NIR laser. Laser power was held constant at 10 W for all tests. Each row corresponds to a different cooking speed (top: 100mm/min, middle: 80 mm/min, bottom: 60 mm/min). All images are cross-sectional cuts showing a side view.*


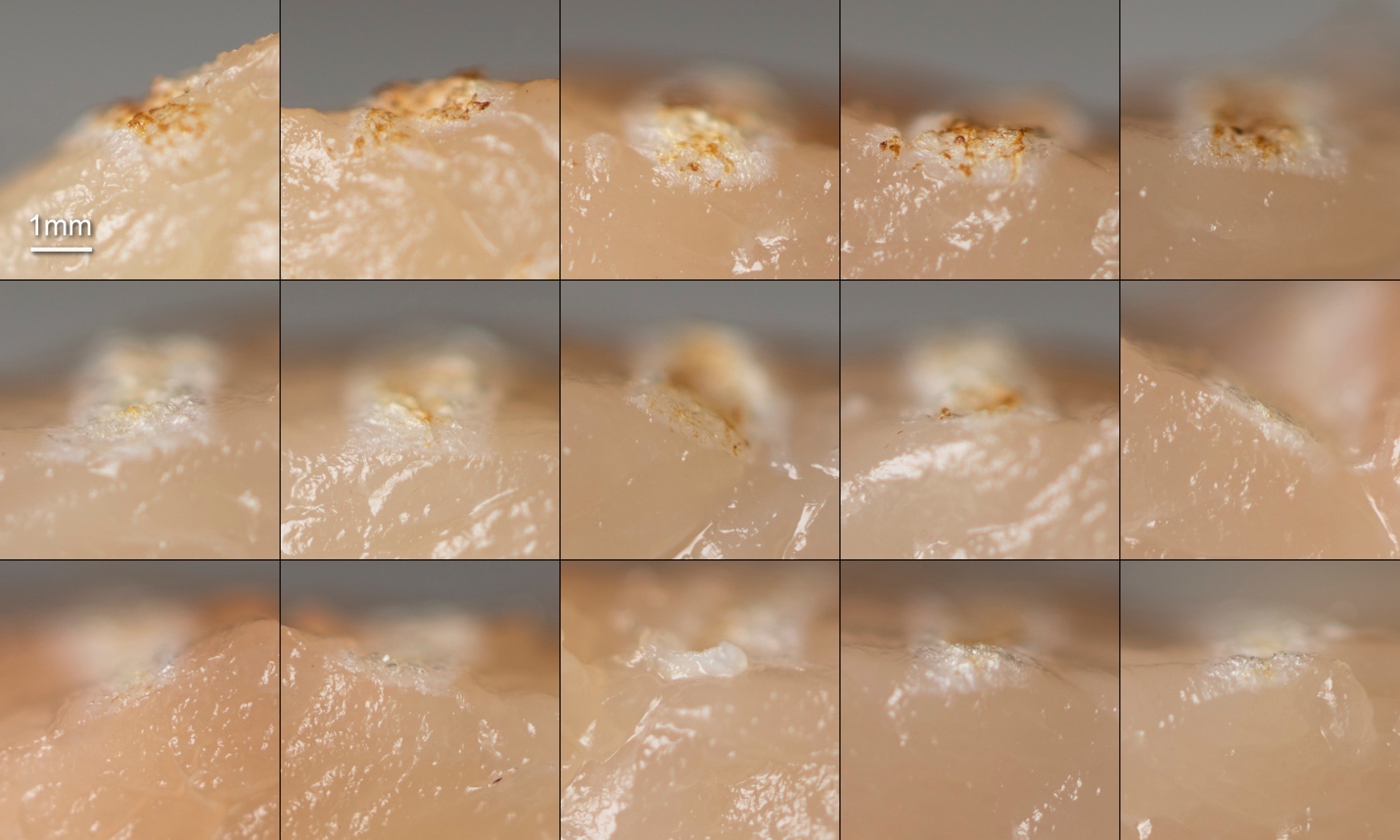
***Fig. S7:*** *Samples used to calculate horizontal and vertical heat-affected zone for chicken cooked via MIR laser. Laser power was held constant at 10 W for all tests. Each row corresponds to a different cooking speed (top: 20mm/s, middle: 30 mm/s, bottom: 40 mm/s). All images are cross-sectional cuts showing a side view.*


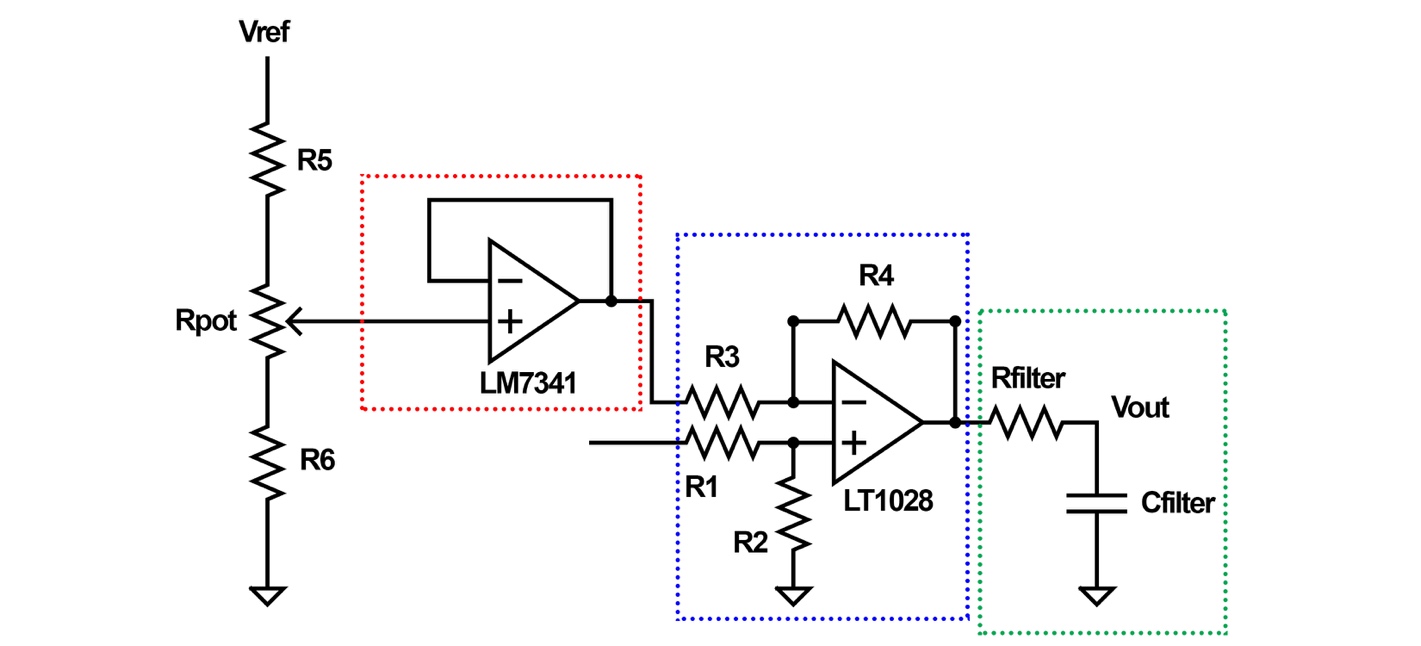


***Fig. S8:*** *Custom amplifier circuit designed with buffer circuit to reduce noise (red-dotted region), a difference amplifier (blue-dotted region), and an RC low-pass filter (green-dotted region). Rfilter = 7.5 kΩ, Cfilter = 100 pF, R1 = 10 kΩ, R2 = 48 kΩ, R3 = 2.1 kΩ, R4 = 10 kΩ, R5 = 47 kΩ, R6 = 4.7 kΩ, Rpot = 10 kΩ.*


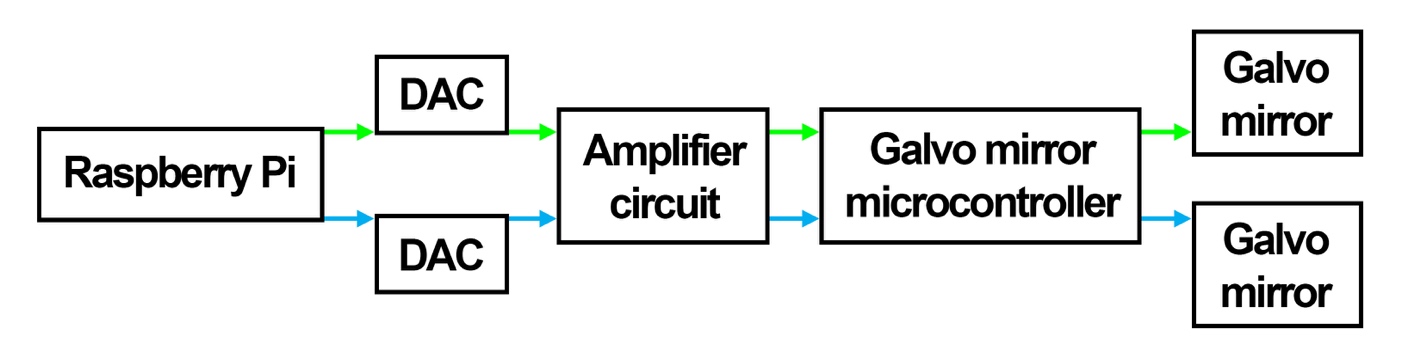


***Fig. S9:*** *Block diagram showing the flow of the signals controlling the x (green arrow) and y (blue arrow) position of the laser via the galvo mirrors.*

*
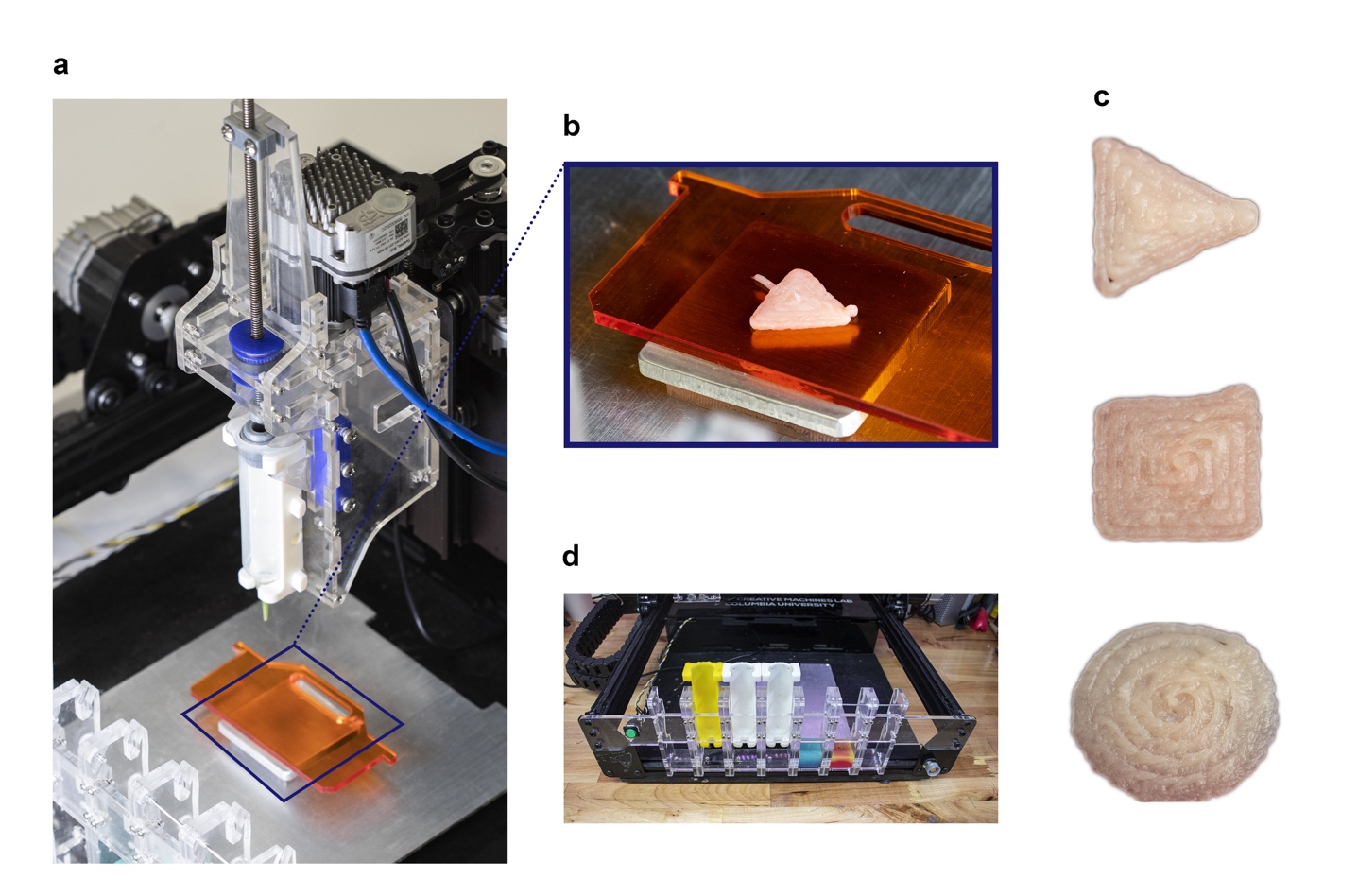
*

***Fig. S10:*** *Mechanism for printing chicken.* ***a*** *Custom fabricated extrusion mechanism that can pick and place material-filled syringes and print them onto a removable platform (orange-tinted tray) for laser cooking.* ***b*** *Close-up shot of printed chicken sample on removable platform (orange-tinted tray).* ***c*** *Examples of single-layer prints (triangle, square, and circle) that were used in the study.* ***d*** *Custom fabricated tool change rack for swapping food cartridges.*


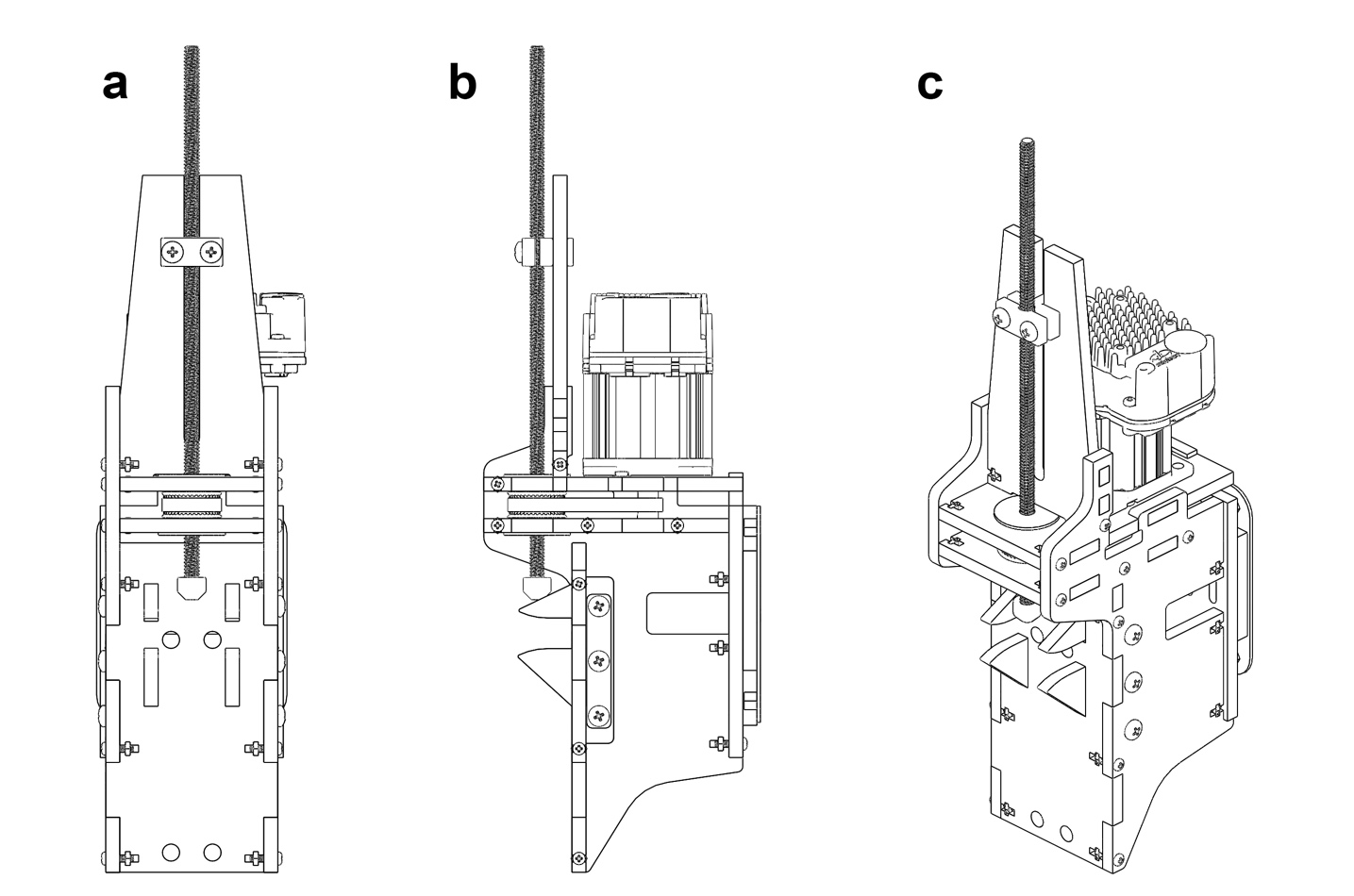


***Fig. S11:*** *Custom designed and fabricated extrusion mechanism. All wall components were laser cut and a few components were 3D-printed.* ***a*** *front view,* ***b*** *side view with side panel removed, and* ***c*** *isometric view.*

**
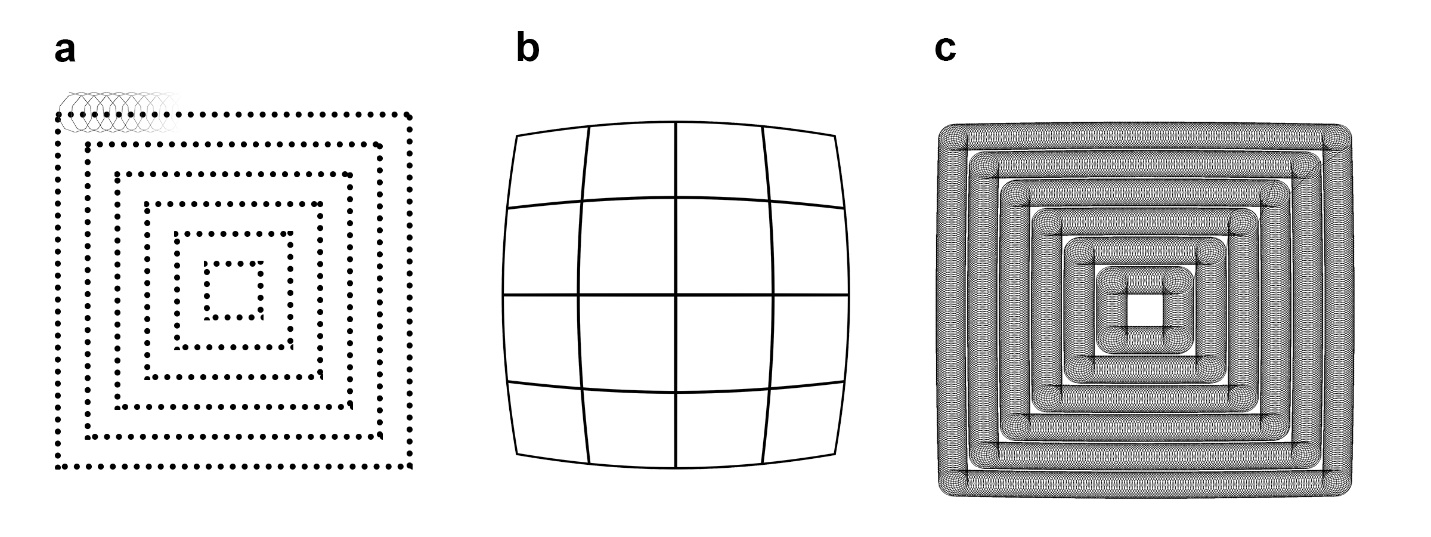
**

***Fig. S12:*** *Design of the laser cooking patterns.* ***a*** *Trochoidal path that follows a series of six square shells (dotted lines) as it propagates along the path.* ***b*** *Barrel distortion was added to the cooking paths to counter the pincushion distortion that resulted from the galvo mirrors.* ***c*** *An example of a cooking pattern used in the trials.*


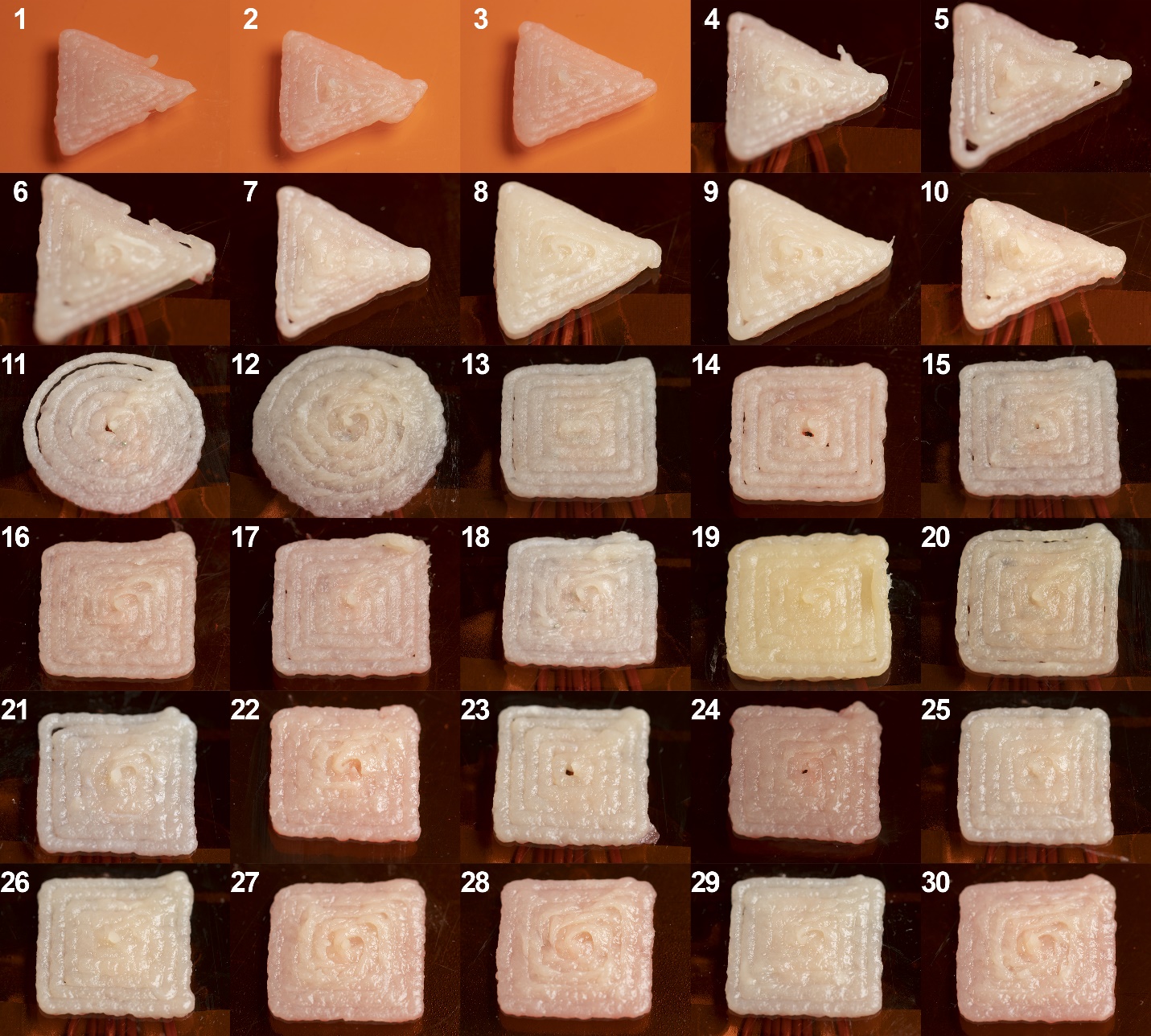


***Fig. S13:*** *Data set of raw printed samples. All samples were used to measure shrinkage and color change. Samples that were used for internal temperature data include 4, 6, 8, 10, 11, 12, 13, 15, 18, 19, 20, 21, 23, 25, 26, 29.*


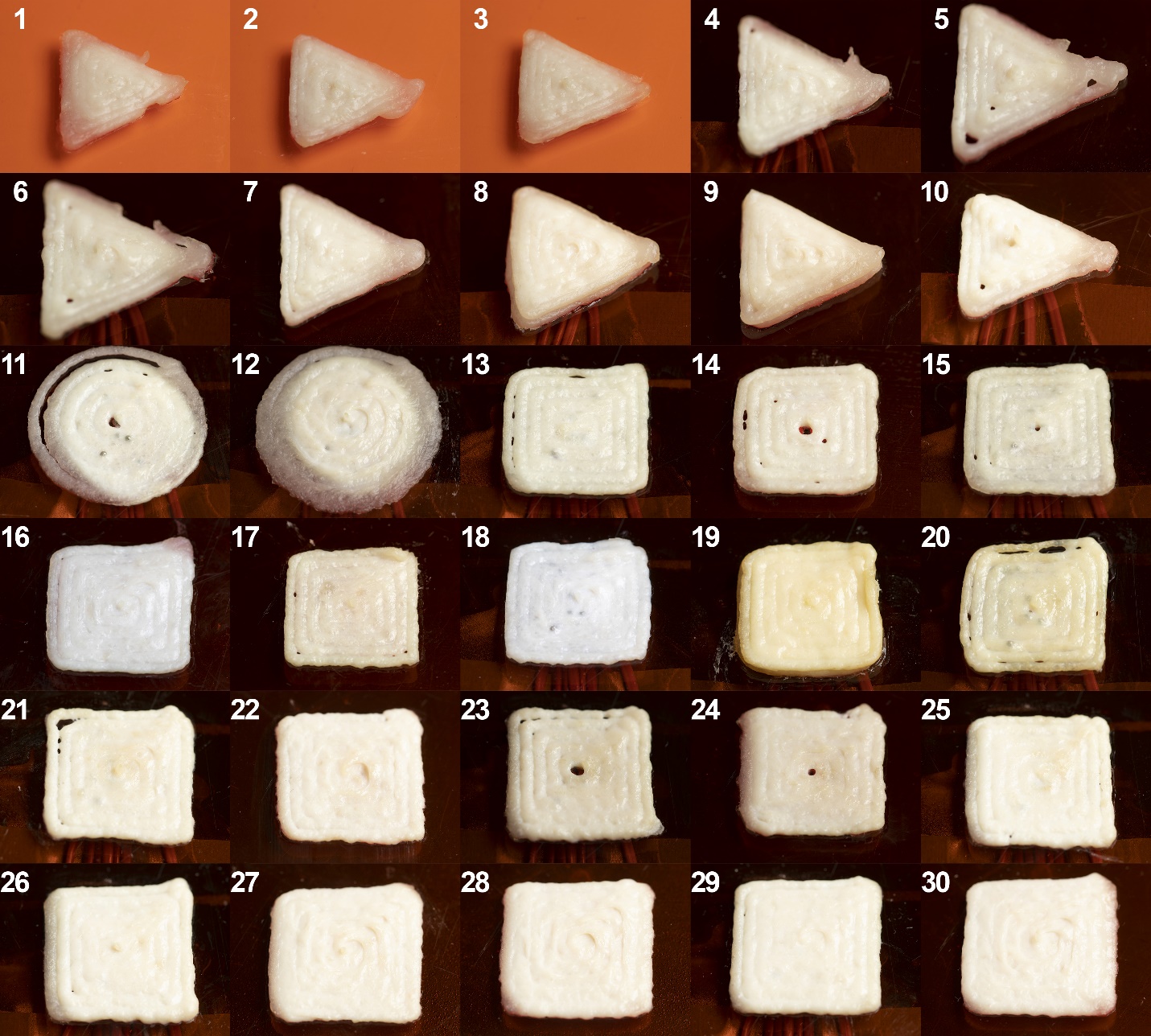


***Fig. S14:*** *Data set of cooked printed samples. All samples were used to measure shrinkage and color change. Samples that were used for internal temperature data include 4, 6, 8, 10, 11, 12, 13, 15, 18, 19, 20, 21, 23, 25, 26, 29.*


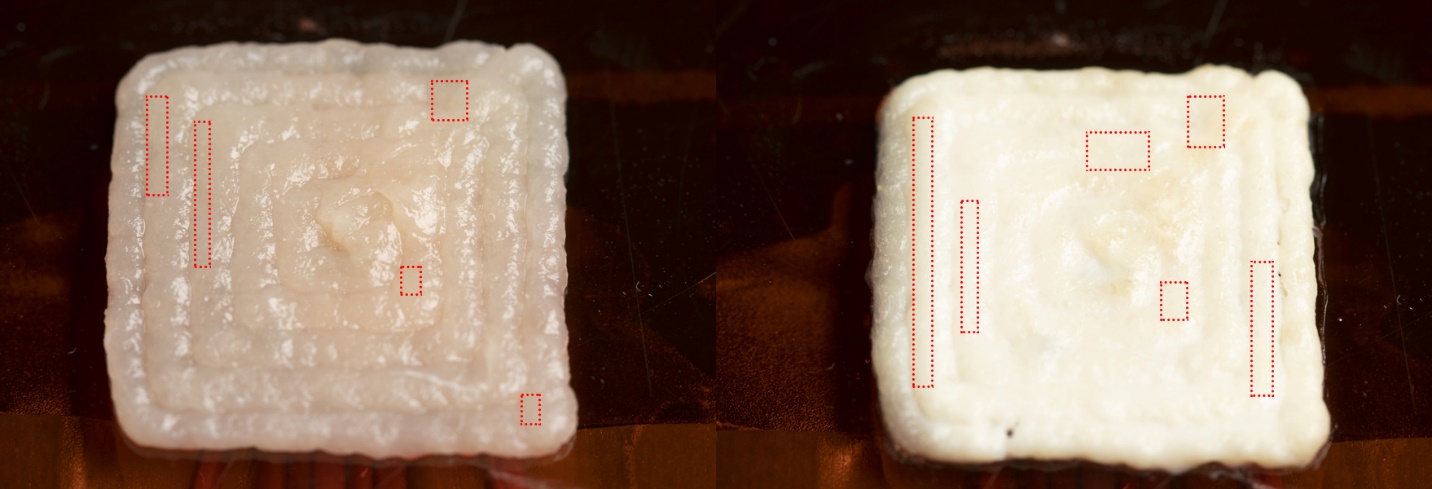


***Fig. S15:*** *Calculating sample color via image analysis. Regions for sampling color on raw (left) and cooked (right) samples are shown with red-dotted regions. Sampling regions were selected based on lowest amount of specular reflectance from the light source. Three regions on each sample were selected and averaged.*


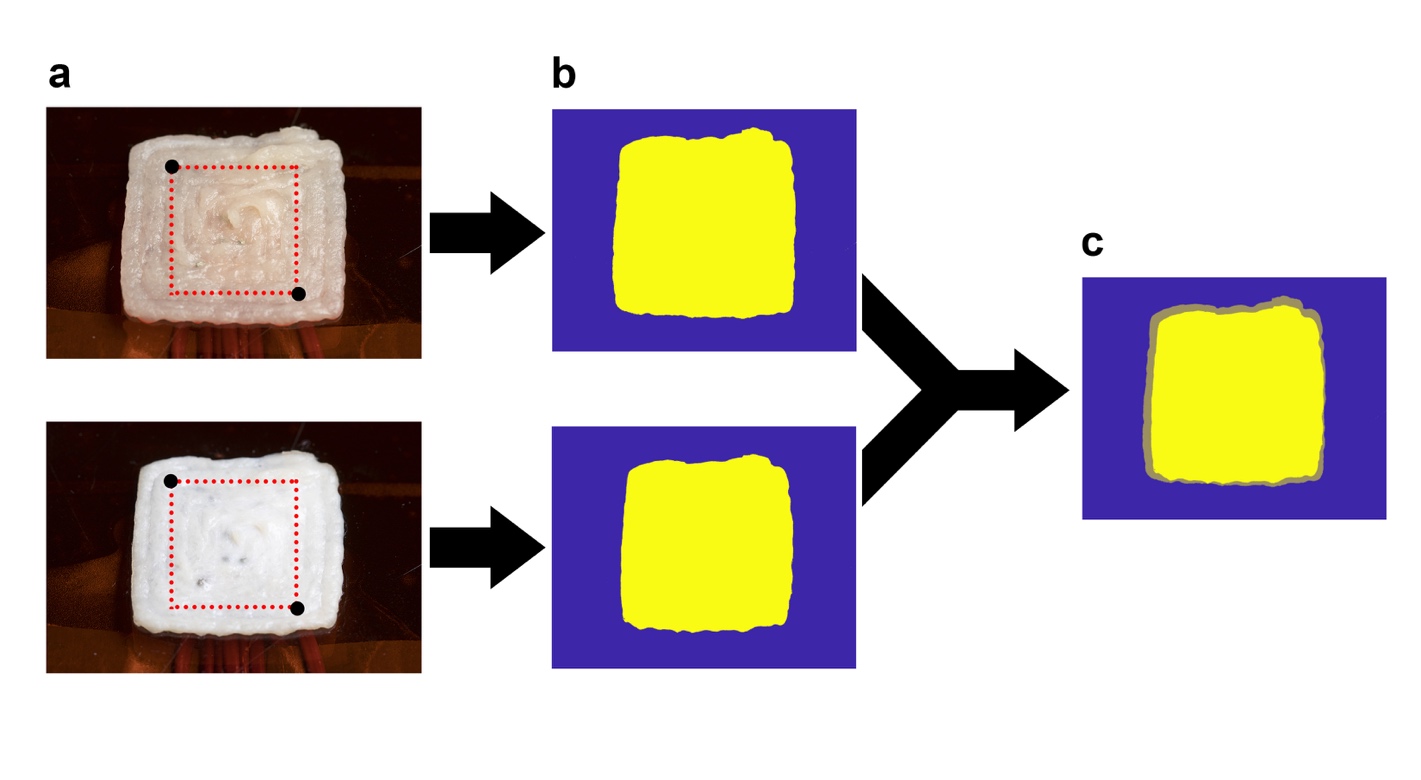


***Fig. S16:*** *Calculating sample shrinkage via image analysis.* ***a*** *Input images are before and after pictures of the raw and cooked chicken, respectively. Area to sample color from is manually selected by the user (denoted by red-dotted square).* ***b*** *Software analyzes image based on selected region and stores all food-containing pixels (yellow).* ***c*** *Shrinkage is calculated by comparing total number of pixels before and after heating.*


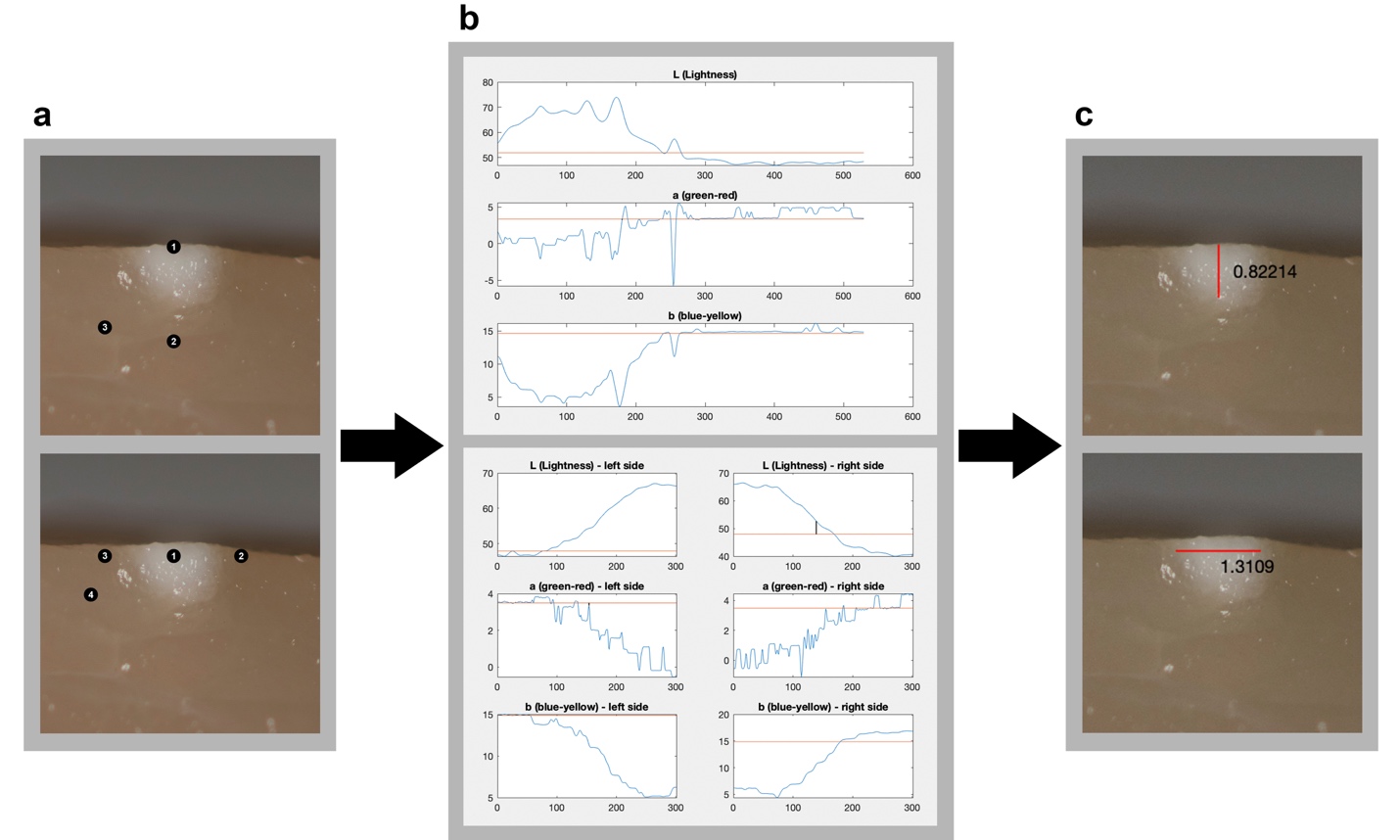


***Fig. S17:*** *Tabulating vertical and horizontal heat-affected zone (HAZ) from laser-cooking trials.* ***a****: To assess vertical HAZ a user needs to select (1) a point at the surface of the sample, (2) a point below the HAZ, and (3) a threshold value to compare “cooked” pixels to. Similarly, to assess horizontal HAZ a user needs to select (1) a point somewhere in the middle of the HAZ, (2) a point to the right of the HAZ, (3) a point to the left of the HAZ, and (4) a threshold value to compare “cooked” pixels to.* ***b****: The shift in color is then plotted and the resulting horizontal and vertical size of the HAZ is calculated.* ***c****: The HAZ is then displayed on the original picture to visually validate each measurement. This process was executed in triplicate for each sample.*

1. Creative Machines Laboratory, Department of Mechanical Engineering, Columbia University in the City of New York, New York, NY, 10027, USA. [↑](#footnote-ref-1)
2. Department of Food Technology, Wageningen University, 6708 PB Wageningen, Netherlands [↑](#footnote-ref-2)
